# Aurora A drives early signalling and vesicle dynamics during T-cell activation

**DOI:** 10.1038/ncomms11389

**Published:** 2016-04-19

**Authors:** Noelia Blas-Rus, Eugenio Bustos-Morán, Ignacio Pérez de Castro, Guillermo de Cárcer, Aldo Borroto, Emilio Camafeita, Inmaculada Jorge, Jesús Vázquez, Balbino Alarcón, Marcos Malumbres, Noa B. Martín-Cófreces, Francisco Sánchez-Madrid

**Affiliations:** 1 Servicio de Inmunología, Hospital Universitario de la Princesa, Instituto Investigación Sanitaria Princesa (IIS-IP), Universidad Autónoma de Madrid, C/ Diego de León 62, Madrid 28006, Spain; 2 Cell-cell Communication Laboratory, Vascular Pathophysiology Area, Centro Nacional Investigaciones Cardiovasculares (CNIC), C/ Melchor Fdz Almagro 3, Madrid 28029, Spain; 3 Cell Division and Cancer Group, Centro Nacional de Investigaciones Oncológicas (CNIO), C/ Melchor Fdz Almagro 3, Madrid 28029, Spain; 4 Centro de Biología Molecular Severo Ochoa, Consejo Superior de Investigaciones Científicas, Universidad Autónoma de Madrid, C/ Nicolás cabrera 1, Madrid 28049, Spain; 5 Laboratory of Cardiovascular Proteomics, Centro Nacional Investigaciones Cardiovasculares (CNIC), C/ Melchor Fdz Almagro 3, Madrid 28029, Spain

## Abstract

Aurora A is a serine/threonine kinase that contributes to the progression of mitosis by inducing microtubule nucleation. Here we have identified an unexpected role for Aurora A kinase in antigen-driven T-cell activation. We find that Aurora A is phosphorylated at the immunological synapse (IS) during TCR-driven cell contact. Inhibition of Aurora A with pharmacological agents or genetic deletion in human or mouse T cells severely disrupts the dynamics of microtubules and CD3ζ-bearing vesicles at the IS. The absence of Aurora A activity also impairs the activation of early signalling molecules downstream of the TCR and the expression of IL-2, CD25 and CD69. Aurora A inhibition causes delocalized clustering of Lck at the IS and decreases phosphorylation levels of tyrosine kinase Lck, thus indicating Aurora A is required for maintaining Lck active. These findings implicate Aurora A in the propagation of the TCR activation signal.

## Abstract (web summary)

Aurora A is a protein kinase that contributes to the progression of mitosis by stimulating microtubule nucleation. Here the authors show that Aurora A also functions during T cell activation by maintaining TCR signaling through Lck activation.

## Graphical abstract

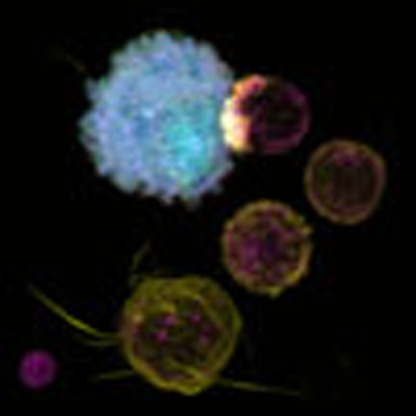

T-cell activation depends on the ability of the T-cell receptor (TCR) to recognize specific antigen peptides presented in the context of the major histocompatibility complex (MHCp) on the antigen-presenting cell (APC)^1^. The binding of the TCR to MHCp promotes the formation of the immune synapse (IS). In this process, the TCR and its associated molecules localize to a central area of the T cell–APC contact, the central supramolecular activating complex (cSMAC). Adhesion molecules relocate to the peripheral SMAC^2^,^3^,^4^. Essential proteins in this process are the Src family kinase members (Lck and Fyn). Lck phosphorylates the immunoreceptor tyrosine-based activation (ITAM) motifs of the TCR/CD3 complex^5^, leading to the recruitment of crucial molecules for the downstream signalling pathways and the IS formation^3^. The formation of the IS also triggers changes in the tubulin cytoskeleton, including the translocation of the centrosome, or microtubule (MT)-organizing centre (MTOC), to the IS, accompanied by the Golgi apparatus, multivesicular bodies and mitochondria^6^,^7^,^8^. These changes facilitate the polarized secretion of cytokines and exosomes towards the APC^9^,^10^,^11^. MTOC polarization orchestrates active MT growth and forms the core of a dense MT network that regulates vesicular traffic at the IS^12^.

The Aurora family of serine/threonine kinases comprises three members in humans—Auroras A, B and C—which are encoded by three different genes^13^ and are key regulators of different mitotic processes^14^. Aurora A plays a critical role in centrosome and spindle dynamics during mitosis, whereas Aurora B regulates the attachment of the kinetochore to MTs and cytokinesis^15^. Aurora A expression and activity peak in late G2 and the protein is concentrated at centrosomes^13^,^16^. During centrosome maturation, Aurora A promotes MT assembly by recruiting nucleation and stabilization factors^17^. Aurora A is self-activated by autophosphorylation at T288 in its T loop, helped by cofactors including Bora, Tpx2, Ajuba and PAK1 (refs 14, 18, 19).

Owing to its role in controlling MT dynamics, we hypothesize that Aurora A may play a role in the activation of T lymphocytes during IS formation. Consistent with our hypothesis, we report here that Aurora A is activated on TCR stimulation and controls the dynamics of MT and CD3ζ vesicles at the IS. We have also found an unexpected contribution of Aurora A to the early and late signalling events in T cells. Specific targeting of Aurora A impairs activation of the TCR/CD3 complex, by deregulating Lck phosphorylation and location, preventing early T-cell activation and downstream expression of CD69, CD25 and interleukin (IL)-2. Our data reveal a novel role for Aurora A as a major regulator of early signalling and the tubulin cytoskeleton during T-cell activation.

## Results

### Active Aurora A localizes to the IS

**Figure 1:**
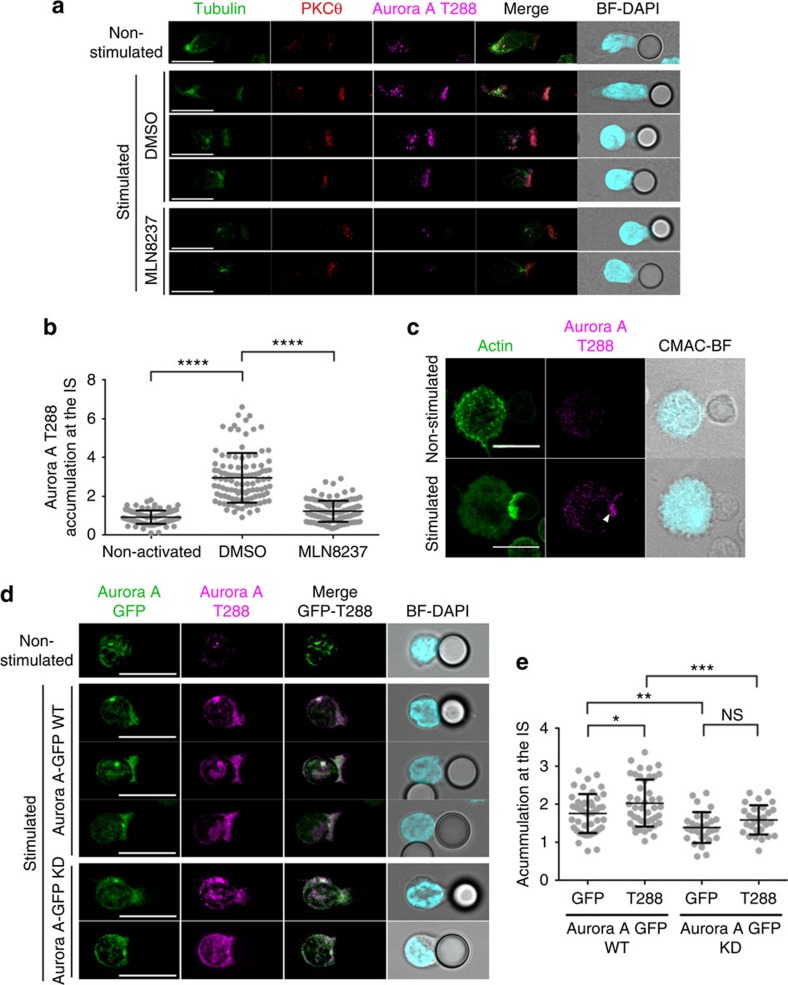
Aurora A is located at the IS contact area and is activated on TCR triggering. (**a**) Maximum *Z* projection of a confocal stack of human primary CD4^+^ T cells pretreated with vehicle (DMSO) or Aurora A inhibitor (MLN8237, 10 μM) and conjugated with anti CD3/CD28-coated beads. Images show three representative conjugates in DMSO and two in MLN8237-treated cells at different stages of cell conjugation. Cells were fixed and stained for PKCθ (red), T288-phosphorylated Aurora A (magenta) and α-tubulin–fluorescein isothiocyanate (FITC) (green). Bright field with DAPI frames are included. Scale bar, 10 μm. (**b**) Quantification of T288-phosphorylated Aurora A accumulation at the IS contact area in conjugates as in **a** from three independent experiments (*n*=93 in non-activated, *n*=105 in DMSO, *n*=109 in MLN8237). Data represent means±s.d. Means were compared with a *t*-test. (**c**) Maximum *Z* projections of confocal stacks of transgenic OTII CD4^+^ cells conjugated with OVA peptide-pulsed bone-marrow-derived dendritic cells (DCs). Cells were incubated for 30 min, fixed and immunostained for T288-phosphorylated Aurora A (magenta) and actin (green). The right-hand image shows CMAC cell tracker labelling of DCs (cyan) and bright field. Scale bar, 10 μm. (**d**) Maximum *Z* projection of a confocal stack of human primary CD4^+^ T cells transfected with Aurora A-GFP WT or Aurora A-GFP KD (green) and conjugated with anti CD3/CD28-coated beads. Cells were incubated for 30 min, fixed and stained for T288-phosphorylated Aurora A (magenta). Bright field with DAPI frames are included. Scale bar, 10 μm. (**e**) Quantification of T288-phosphorylated Aurora A and transfected Aurora A accumulation at the IS contact area in conjugates as in **d** (*n*=45 in Aurora A-GFP WT, *n*=29 in Aurora A-GFP KD). Data represent means±s.d. Means were compared with a *t*-test. n.s., nonsignificant. *P<0.05, **P<0.01, ***P<0.001, ****P<0.0001.

To assess the specific location of activated Aurora A, we conjugated human CD4^+^ T cells from peripheral blood from healthy donors with beads coated with stimulatory anti-CD3 and anti-CD28 antibodies, and stained with anti-phospho-specific antibody against the Aurora-T288 residue, which detects active Aurora A. In these experiments, T288-phosphorylated endogenous Aurora A was found in two different pools: one in the centrosome and the other at the T-cell-bead contact region (examples of conjugates at different stages of the process are shown; Fig. 1a); the low signal of activated Aurora A in non-stimulated control conjugates was not detected at the IS (Fig. 1a). Pretreatment of peripheral-blood-derived human CD4^+^ T cells with the specific Aurora A inhibitor MLN8237 blocked the phosphorylation of Aurora A (Fig. 1a). Quantitative analyses showed that phosphorylated Aurora A is accumulated at the IS in stimulated CD4^+^ T cells, and that this is prevented by MLN8237 treatment (Fig. 1b). Staining of phosphorylated endogenous Aurora A on TCR stimulation was also abolished in T cells silenced with specific small interfering RNAs (siRNAs) for Aurora A, confirming the specific binding of the antibody (Supplementary Fig. 1a). Active Aurora A also localized at the IS in conjugates of naive mouse OTII T lymphocytes with primary dendritic cells pulsed with OVA peptide (Fig. 1c). These results clearly show that TCR triggering promotes the activation of Aurora A and its recruitment to the IS. However, pretreatment of J77 cells with the specific Aurora A inhibitor MLN8237 did not alter the number of conjugates formed with staphylococcal enterotoxin E (SEE)-pulsed Raji cells (Supplementary Fig. 1b), indicating that inhibition of Aurora A does not result in a global defect in cytoskeleton dynamics.

To parse the localization of activated Aurora A with respect to total Aurora A, we transfected primary CD4^+^ T cells with Aurora A-GFP wild type (WT) or Aurora A-GFP KD (kinase dead mutant) and then conjugated the transfected cells with stimulatory anti-CD3/CD28-coated beads (Fig. 1d,e). Quantitative analysis of Aurora A-GFP or active Aurora A (Aurora A T288) accumulation demonstrated that it is mainly found at the IS. However, Aurora A KD accumulation at the IS is significantly decreased, compared with WT. Moreover, overexpression of the Aurora A-GFP KD mutant, disperses the remaining active protein. Thus, the phosphorylated active form of Aurora A is specifically recruited to the IS.

### Aurora A controls MT dynamics at the IS

**Figure 2:**
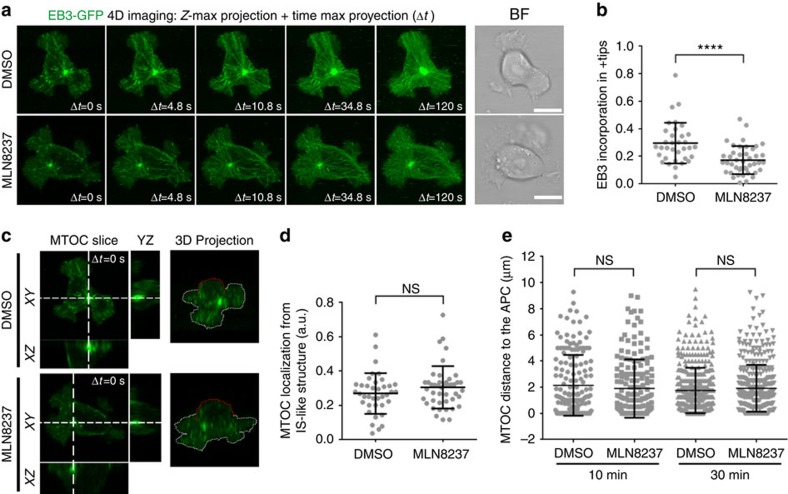
MT dynamics at the IS is impaired by Aurora A chemical inhibition. (**a**–**d**) Imaging of EB3-GFP-expressing CH7C17 T cells (pretreated with DMSO or MLN8237 and settled on corresponding anti-CD3/CD28-coated glass-bottom chambers). Maximal projection of *XYZ* stacks for fluorescence and single bright-field (BF) images are shown. Scale bar, 10 μm. (**b**) Ratio of EB3-GFP fluorescence incorporated in +tips from *XYZ* stacks (0 s; *n*=34 in DMSO and *n*=43 in MLN8237). Data represent means±s.d. Means were compared with a Mann–Whitney test. (**c**) Orthogonal and 3D projections from *XYZ* stacks. Dotted white or red lines indicate contact with substrate or media, respectively. (**d**) Ratio of the MTOC location from the IS-like structure (*n*=38 in DMSO, *n*=44 in MLN8237). (**e**) Distance from the T-cell MTOC to the APC contact area in conjugates of T cells with SEE-pulsed APCs (10 min, *n*=166 in DMSO, *n*=168 in MLN8237; 30 min, *n*=412 in DMSO, *n*=394 in MLN8237). Data represent means±s.d. from three independent experiments. Means were compared with a *t*-test.

Aurora A plays an important role in the dynamics of the centrosome during mitosis^20^,^21^. To ascertain its possible function in MT dynamics and centrosomal polarity during T-cell activation, we analysed the dynamics of the microtubular network in CH7C17 T cells transiently transfected or stably expressing an EB3-GFP fusion protein (EB3 cells; Fig. 2a–d and Supplementary Movie 1). EB3 and EB1 (end-binding proteins) are plus-tip-tracking proteins that are also found in the pericentrosomal matrix and promote MT growth^22^. Cells were settled on anti-CD3/CD28-coated chambers and time-lapse confocal three-dimensional (3D) imaging was performed by *XYZ* stack acquisition. The stimulating surface allows IS-like formation, associated centrosome polarization and MT polymerization^12^. EB3 cells were pretreated or not (vehicle) with MLN8237 for 30 min before imaging. Maximal projection of the *XYZ* stack (Fig. 2a and Supplementary Movie 1) revealed that the relative amount of EB3-GFP incorporated into MT plus ends (+tips) was clearly decreased in Aurora A-inhibited cells. This effect was measured in 3D as the ratio of EB3-GFP fluorescence incorporated in +tips with respect to the whole-cell fluorescence using Imaris software, confirming that Aurora A-inhibited cells polymerize MTs less efficiently (Fig. 2b and Supplementary Movie 1). The amount of polymerized MT observed along the time course was clearly decreased in MLN8237-treated cells (Fig. 2a). We also analysed the localization of the MTOC and the EB3-GFP fluorescence by 3D and orthogonal projections of the *XYZ* stacks. Fluorescence was mainly detected close to the stimulating surface (Fig. 2c,d). This can be also observed by comparing bottom and top slices of the *XYZ* stacks (Supplementary Fig. 2a). Despite the effect of Aurora A inhibition on MT dynamics, no significant change on MTOC translocation in cell conjugates was observed either at 10 or at 30 min of activation (Fig. 2e).

**Figure 3:**
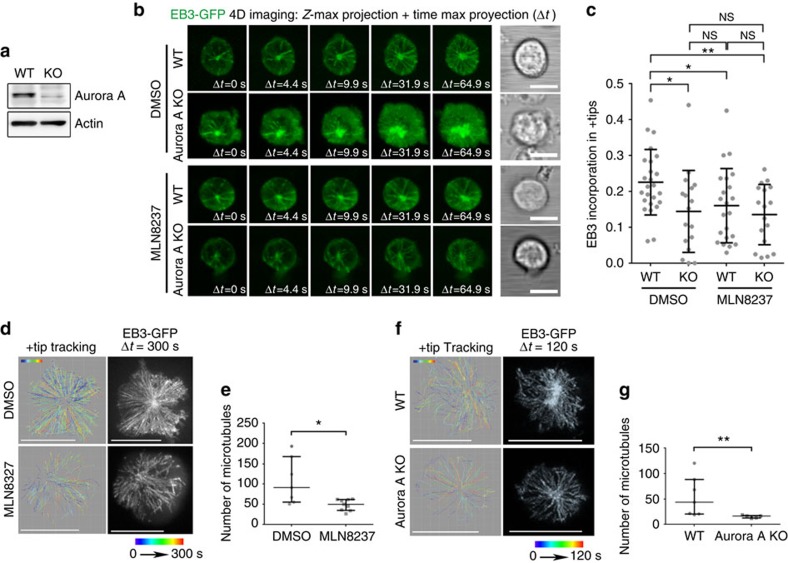
Aurora A gene ablation impairs MT dynamics at the IS. (**a**) Immunoblot analysis of Aurora A protein expression in CD4^+^ T cells WT and KO. (**b**,**c**) Imaging of EB3-GFP-expressing Aurora-A-deficient and control CD4^+^ T cells, pretreated with DMSO or MLN8237 and settled on corresponding anti-CD3/CD28-coated glass-bottom chambers. Maximal projection of *XYZ* stacks for fluorescence and single bright-field (BF) images are shown. Scale bar, 5 μm. (**c**) Ratio of EB3-GFP fluorescence incorporated in +tips from *XYZ* stack (0 s, *n*=25 in WT, *n*=17 in KO, *n*=22 in WT MLN8237 and *n*=17 in KO MLN823). Data represent means±s.d. Means were compared with a Mann–Whitney test. Map of the trajectories of EB3-GFP-decorated MT plus tips in human CH7C17 T cells pretreated with DMSO or MLN8237 (**d**,**e**), or in Aurora-A-deficient and control CD4^+^ T cells (**f**,**g**) and settled on anti-CD3/CD28-coated glass-bottom chambers. Images were taken every 300 ms under a TIRF microscope at a penetrance of 150 nm. MT +tips were tracked with Imaris software over 5 (**d**) or 2 min (**f**). Maximal projections of the time lapse from representative cells are shown. Scale bar, 10 μm. (**e**,**g**) Quantification of the number of MT plus tip tracks presented in **d** (**e**; *n*=7 in DMSO, *n*=9 in MLN8237) and **g** (**f**; *n*=6). Error bars represent interquartile rage. Medians were compared with a Mann–Whitney test. n.s., nonsignificant. *P<0.05, **P<0.01, ***P<0.001, ****P<0.0001.

To further assess the function of Aurora A in primary naive T cells, we used a mouse model of conditional Aurora deficiency. CD4^+^ cells were isolated from lymph nodes and spleens of experimental [Aurka(lox/lox); RERT(ert/ert)] (knockout (KO) mice) and control [Aurka(+/+); RERT(ert/ert)] (WT mice), treated with tamoxifen and IL-7 for 96 h, to suppress Aurora A expression (Fig. 3a). These cells were transfected with a plasmid encoding EB3-GFP and then activated with anti-CD3/CD28 stimulating monoclonal antibodies (Fig. 3b,c and Supplementary Movie 2). We found that Aurora A-deficient T cells had significantly less EB3 incorporation in MT +tips than their WT counterparts (Fig. 3b,c and Supplementary Movie 2). Furthermore, the effect of Aurora A deficiency was similar to the effect of the MLN8237 inhibitor on WT cells, whereas the inhibitor did not have additional effects on Aurora A KO cells, suggesting that these and previously recorded effects of the inhibitor were Aurora A specific. MTOC and EB3-GFP tracking of MTs was also observed at the bottom of the cells (Supplementary Fig. 2b,c).

We next tracked the dynamics of MT growth using EB3-GFP imaging and total internal reflection fluorescence (TIRF) microscopy in cells settled on anti-CD3/CD28-coated surfaces, to improve the *XY* spatial and time resolution^23^,^24^,^25^. EB3 cells were treated with MLN8237 or dimethyl sulfoxide (DMSO; vehicle) for 30 min before imaging and images were taken every 300 ms. MLN8237-treated EB3-GFP cells had fewer EB3-decorated tips emerging from the centrosome, indicating impaired MT growth (Fig. 3d,e and Supplementary Movies 3 and 4). MT growth was similarly impaired in Aurora-KO primary CD4^+^ T cells, displaying fewer and slower growing MTs than control cells (0.140±0.037 and 0.190±0.023 μm s^−1^, respectively; mean±s.d.) (Fig. 3f,g and Supplementary Movies 5 and 6). Thus, these results show that the MT network at the IS is disrupted in T cells with defective Aurora A activation.

### Aurora A regulates CD3ζ-bearing vesicles traffic at the IS

**Figure 4:**
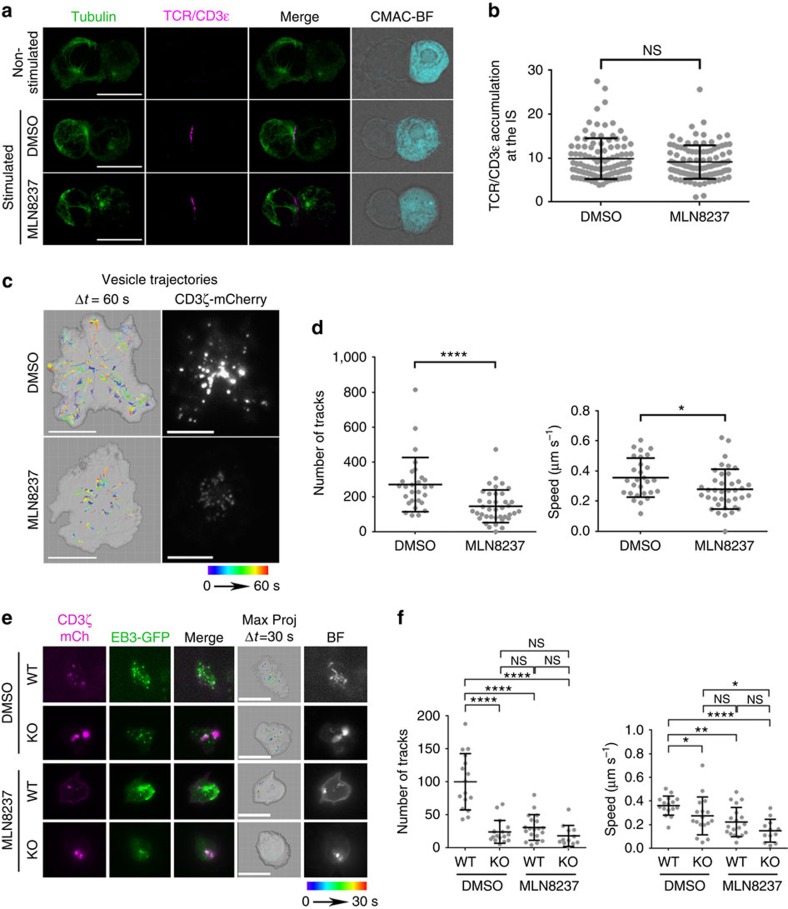
Trafficking of CD3-bearing vesicles at the IS is impaired by Aurora A chemical inhibition or gene ablation. (**a**) Maximum *Z* projections of confocal stacks of Jurkat T cells pretreated with vehicle (DMSO) or Aurora A inhibitor (MLN8237) and conjugated with SEE-pulsed Raji B cells. Cells were incubated for 30 min, fixed and stained for α-tubulin (green) and TRC/CD3ɛ (magenta). The right-hand image shows CMAC cell tracker labelling of Raji B cells (cyan) and bright field. Scale bar, 10 μm. (**b**) Graph shows quantification of TRC/CD3ɛ clustering at the IS from as in **a**. Means±s.d. is shown; *t*-test was used to compare means (*n*=101 in DMSO and in MLN8237). Map of the trajectories of CD3ζ-cherry-bearing vesicles in human CH7C17 T cells (**c**) or Aurora-A-deficient and control CD4^+^ T cells (**e**) pretreated with vehicle (DMSO) or MLN8237 inhibitor and settled on corresponding anti-human or anti-mouse stimulating anti-CD3/CD28-coated glass-bottom chambers. Images were taken every 100 (**c**) or 110 ms (**e**) under a TIRF microscope at a penetrance of 200 nm with 561 nm laser; vesicles were tracked with Imaris software over 60 (**c**) or 30 s (**e**) and maximal projections of the time lapse are shown for tracks. A representative cell is shown for each case. Fluorescence images from CD3ζ-mCherry (**c**,**e**) and EB3-GFP are also shown (**e**). (**d**,**f**) Quantification of the number of vesicle tracks and the speed of vesicles from cells analysed in **c** and **e** from three independent experiments (**d**, *n*=28 in DMSO, *n*=39 in MLN8237; **f**, *n*=16 in WT, *n*=17 in KO, *n*=19 in WT MLN8237, *n*=12 in KO MLN8237). Data represent means±s.d. Means were compared with a Mann–Whitney test. n.s., nonsignificant. *P<0.05, **P<0.01, ***P<0.001, ****P<0.0001.

The impaired MT growth observed in Aurora A-targeted T cells did not affect the localization of the surface TCR/CD3 complexes at the IS, as TCR/CD3*ɛ* was comparably clustered at the IS of untreated and MLN8237-treated T-cell conjugates with APC (Fig. 4a,b). We next assessed the dynamics of CD3ζ-bearing vesicles at the IS. CD3ζ traffics through endosomal compartments towards the IS^26^. These vesicles move associated to MTs and support the sustained activation of the T cell at the IS^12^,^27^. The vesicles enter and leave the TIRF plane, some of them moving towards the position of the centrosome at the centre of the IS-like structure, probably along the MTs. Jurkat T cells expressing CD3ζ-mCherry were treated with DMSO or MLN8237, settled onto anti-CD3/CD28 and analysed by TIRF microscopy. Images were taken every 100 ms (200 nm penetrance) and the trajectories of detected vesicles were tracked. Treatment with MLN8237 decreased the number of vesicles at the IS-like structure and disrupted the movement of those that were present (Fig. 4c,d and Supplementary Movies 7 and 8). Therefore, the effect of Aurora A inhibition on MT dynamics impedes the movement of vesicles towards the IS structure, a finding confirmed by the reduced speed of vesicles in Aurora A-inhibited cells (Fig. 4d). A similar phenotype was observed in Aurora KO cells, with few or no vesicles moving towards the centre of the IS-like structure. Treatment of WT cells with the Aurora A inhibitor caused a similar effect to Aurora A-deficiency (Fig. 4e,f and Supplementary Movie 9).

### Aurora A blockade does not affect TCR-driven actin dynamics

**Figure 5:**
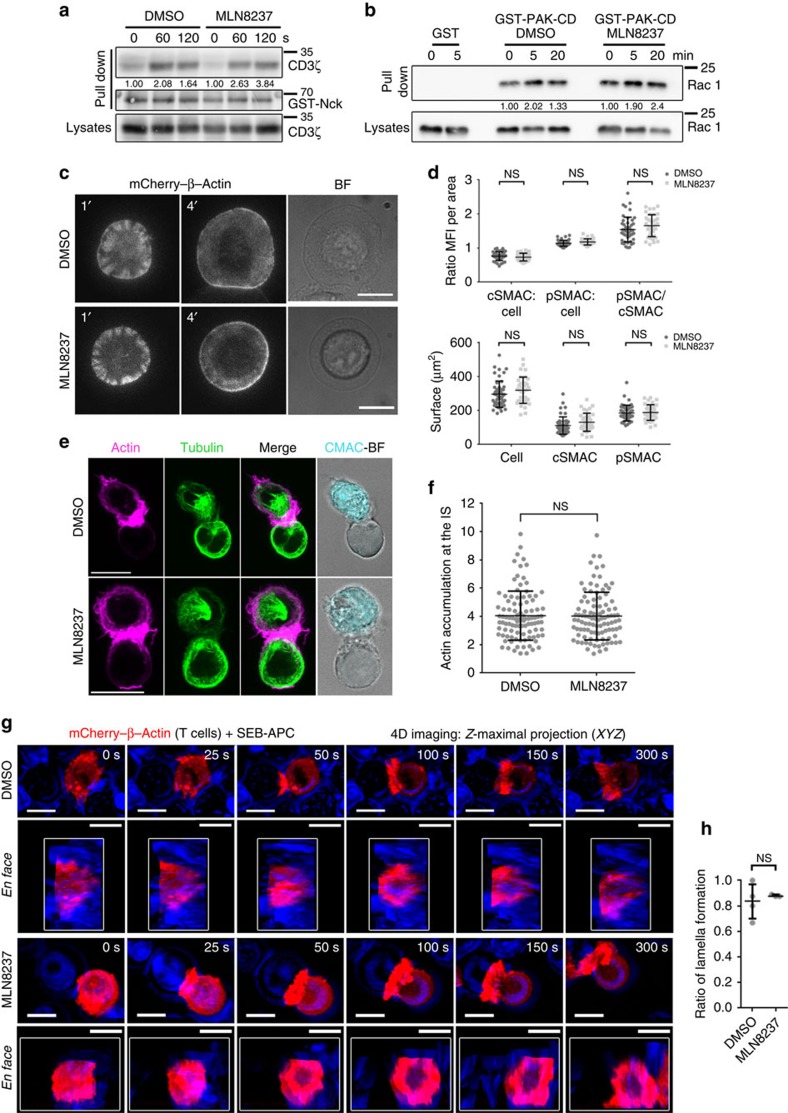
Aurora A inhibition does not affect actin cytoskeleton dynamics. (**a**) Immunoblot of a pull-down assay of GST-Nck fusion protein from cell lysates of control (DMSO; vehicle) or Aurora A inhibitor (MLN8237)-pretreated human T lymphoblasts. Activation was performed with soluble anti-CD3ɛ antibodies for indicated times. CD3ζ and GST are shown. CD3ζ content in whole-cell lysates is indicated in the bottom row. (**b**) Immunoblotting of Rac1 pull-down assay of GST and GST-PAK-CD from cell lysates of DMSO- or MLN8237-pretreated Jurkat T cells activated with SEE-pulsed Raji B cells (APCs) for the indicated times. Loading control for Rac1 in whole-cell lysates is shown. (**c**) Images from TIRFm time-lapse analysis of mCherry–β-actin-expressing Jurkat T cells spreading over anti-CD3/CD28-coated glass-bottom chambers. Cells were pretreated with DMSO or MLN8237. Images were taken every 100 ms for 5 min at 90 nm penetrance. A corresponding bright-field image is shown. Scale bar, 10 μm. (**d**) Quantification of the area occupied by the whole cell (lamella), the actin-rich area (peripheral SMAC (pSMAC)), the central area (cSMAC) and the distribution of mean fluorescence intensity per area (ratios cSMAC:cell; pSMAC:cell and cSMAC/pSMAC) from cells in **c** (*n*=48 and *n*=36, three independent experiments). Cells were fixed after spreading (4 min) and fluorescence images were taken. Data represent means±s.d.; *t*-test. n.s., nonsignificant. (**e**) Maximum *Z* projections of confocal stacks from DMSO- or MLN8237-pretreated Jurkat T cells conjugated with SEE-APCs. Cells were incubated for 30 min, fixed and stained for α-tubulin (green) or actin (magenta). The right-hand image shows CMAC cell tracker labelling of APCs (cyan) and bright field. Scale bar, 10 μm. (**f**) Quantification of actin accumulation at the IS contact area in conjugates as in **e** from three independent experiments (*n*=100). Data represent means±s.d.; *t*-test. (**g**) Image sequence for IS formation between mCherry–β-actin-expressing T cells and SEB-APCs (DMSO- or MLN8237-treated). *XYZ* stacks were acquired every 25 s (maximal projections of *XYZ* stacks and 3D reconstructions with Imaris Software are shown from representative conjugates). (**h**) Ratio of T cells forming lamella on contact with an APC from **g**. Data represent median±interquartile range. Mann–Whitney test (DMSO: 28 cells (*n*=4); MLN8237: 25 cells (*n*=3)).

To further analyse the role of Aurora A in the control of cytoskeletal dynamics at the IS, we assessed the effect of Aurora A inhibition on the activation-dependent interaction of TCR/CD3 with the actin–cytoskeleton-associated protein Nck. This interaction is enabled by the conformational change in the TCR/CD3*ɛ* complex on antigenic triggering^28^. Aurora A inhibition had no effect on CD3ζ–Nck association in pull-down assays (Fig. 5a). This is in agreement with a surface recruitment and accumulation of TCR/CD3*ɛ* to the IS in Aurora A-inhibited cells (Fig. 4a,b). Using a similar approach, we assessed whether Aurora A impairment affects the activation of the small GTPase Rac1, a hallmark for TCR-dependent actin polymerization^29^. Likewise, no effect was detected in Rac1 pull-down assays with the GST-PAK-CD (p21-activated kinase CRIB Domain^30^) in stimulated CD4^+^ T cells when using MLN8327 inhibitor (Fig. 5b). Furthermore, the Aurora A inhibitor did not affect the spreading of mCherry–β-actin-expressing T cells on anti-CD3/CD28-coated coverslips, measured either as the total occupied surface or as the rate of membrane extension on the coverslip (Fig. 5c,d). This finding correlated with a similar distribution of mCherry–β-actin at the peripheral SMAC and cSMAC in control and Aurora A-inhibited cells. Aurora A inhibition also had no effect on the total area occupied by adhered cells or their lamellae (Fig. 5d). Similarly, actin accumulation at the IS in T cell–APC conjugates was not significantly affected by inhibition of Aurora A (Fig. 5e,f). We therefore analysed the formation of the actin ring in cell conjugates using time-lapse 3D confocal imaging. Actin accumulation and ring formation was similar in control and MLN8237-treated cells (Fig 5g,h and Supplementary Movie 10). Therefore, Aurora A appears to specifically affect the tubulin cytoskeleton at the IS, without affecting actin-based dynamics.

### Aurora A inhibition impairs early TCR signalling

**Figure 6:**
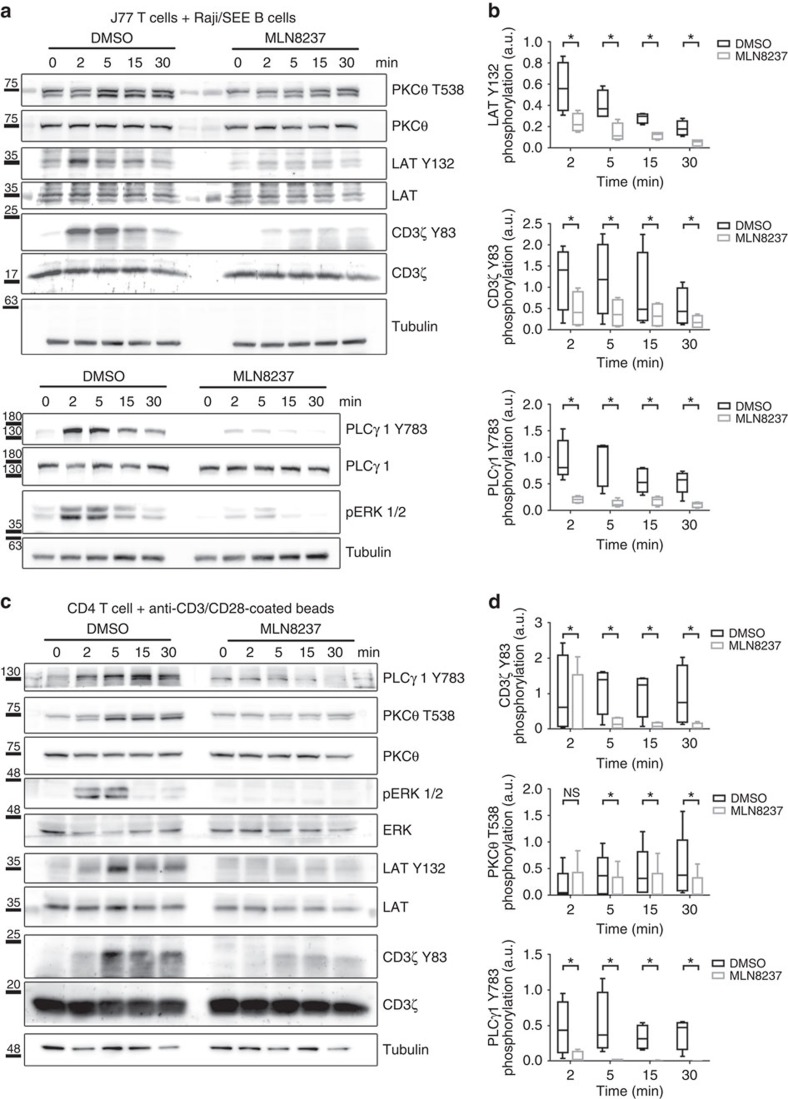
Aurora A inhibition impairs TCR signalling pathways. (**a**) Immunoblottings showing phosphorylation of the indicated molecules in lysates of J77 Jurkat T cells pretreated with vehicle (DMSO) or Aurora A inhibitor (MLN8237) and conjugated for the indicated times with SEE-pulsed Raji B cells. (**b**) Quantification of blots as in **a** from four to six independent experiments. Error bars represent interquartile range. Medians were compared with a Friedman test (*P<0.05). n.s., nonsignificant. (**c**) Immunoblottings showing phosphorylation of the indicated molecules in lysates of DMSO- or MLN8237-pretreated primary human CD4^+^ T cells conjugated for the indicated times with anti-CD3/CD28-coated beads. (**d**) Quantification of blottings as in **c** from four to six independent experiments. Error bars represent interquartile range. Medians were compared with a Friedman test (*P<0.05). n.s., nonsignificant.

**Figure 7:**
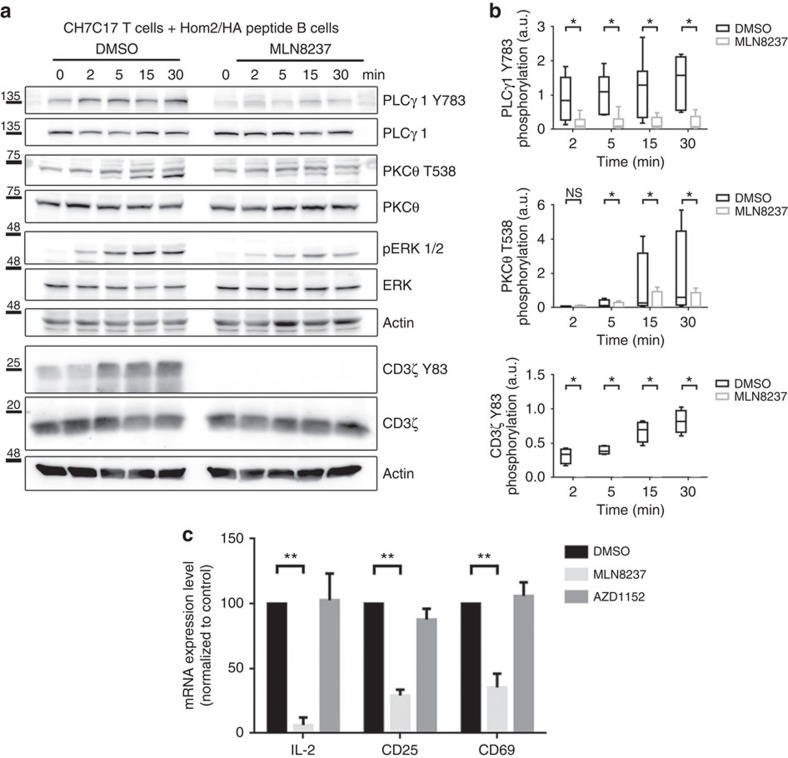
Aurora A inhibition impairs TCR signalling and gene expression. (**a**) Immunoblottings showing phosphorylation of the molecules indicated in lysates of CH7C17 Jurkat T cells pretreated with DMSO or MLN8237 and conjugated for the indicated times with HA-peptide-pulsed Hom2 B cells. (**b**) Quantification of blots as in **a**–**c** from four to six independent experiments. Error bars represent interquartile range. Medians were compared with a Friedman test (*P<0.05). n.s., nonsignificant. (**c**) *IL2*, *CD69* and *CD25* mRNA levels in primary human CD4^+^ T cells pretreated with DMSO, MLN8237 (10 μM) or the Aurora B inhibitor AZD1152 (100 nM) and activated by settling on anti-CD3/CD28-coated plates for 4 h. mRNA levels were normalized to the housekeeping gene *GAPDH* and the levels of the target mRNA in non-stimulated cell levels. Error bars represent interquartile rage. Medians were compared with a Mann–Whitney test. **P<0.01.

To assess the possible role of Aurora A in TCR signalling, we analysed the phosphorylation of several canonical downstream molecules that are phosphorylated in response to cognate interactions in SEE-stimulated Jurkat T cells (Fig. 6a,b) and anti-CD3/CD28-stimulated human primary CD4^+^ T cells (Fig. 6c,d). The phosphorylation of specific residues in CD3ζ (Y83), LAT (Linker for Activation of T cell; Y132), PLCγ1 (Phospholipase C; Y783), PKCθ (T538) and ERK1/2 (T202/Y204) was greatly diminished on Aurora A inhibition with MLN8237. The role of Aurora A in TCR signalling was also confirmed in an MHC/peptide-specific system, in which MLN8237-treated CH7C17 Jurkat T cells were stimulated with Hom2 lymphoblastoid B cells preloaded with haemagglutinin (HA) peptide (Fig. 7a,b). The effect of Aurora A inhibition on TCR downstream signalling was dose dependent (Supplementary Fig. 3a,b). As a control of MLN8237 specificity, we added the inhibitor just before the activation of T cells and the same effect was observed (Supplementary Fig. 3c). By extensively washing the inhibitor before activation, the phosphorylation levels of these specific residues were restored, indicating that the effects of the inhibitor were reversible (Supplementary Fig. 3d). MLN8237 shows a 200-fold higher selectivity for Aurora A over Aurora B^31^; nonetheless, to rule out a possible role of Aurora B, we treated J77 T cells with AZD1152 (100 nM), which is 3,700 times more selective for Aurora B^32^. AZD1152 had no effect on the phosphorylation of T-cell proteins (Supplementary Fig. 4), confirming that proper T-cell activation critically depends of the isoform A, but not B, of Aurora kinase. This was further confirmed in conjugates of Aurora-A-silenced Jurkat T cells and Staphylococcal enterotoxin B (SEB)-preloaded Hom2 B cells as APCs. The activation of CD3ζ-dependent molecules was defective in Aurora-A-silenced cells, with below-normal LAT phosphorylation on residue Y132, probably responsible for the concomitant decreases in PLCγ1 (Y783) and PKCθ (T538) phosphorylation (Supplementary Fig. 5a).

To determine the role of Aurora A in late events of T-cell activation, we examined the messenger RNA expression of IL-2, CD25 and CD69. Human CD4^+^ T lymphocytes were treated with MLN8237 and AZD1152 or vehicle for 30 min, and stimulated with anti-CD3/CD28 antibodies for 3 h. Inhibition of Aurora A impaired the upregulation of IL-2, CD25 and CD69 mRNA determined by reverse transcriptase–PCR (Fig. 7c), indicating a defect in late T-cell activation. In contrast, Aurora B inhibition had no effect on the mRNA production of these genes, supporting a specific role for Aurora A and its regulated pathways in T-cell activation.

### TCR signalling is impaired in Aurora-A-deficient mice

**Figure 8:**
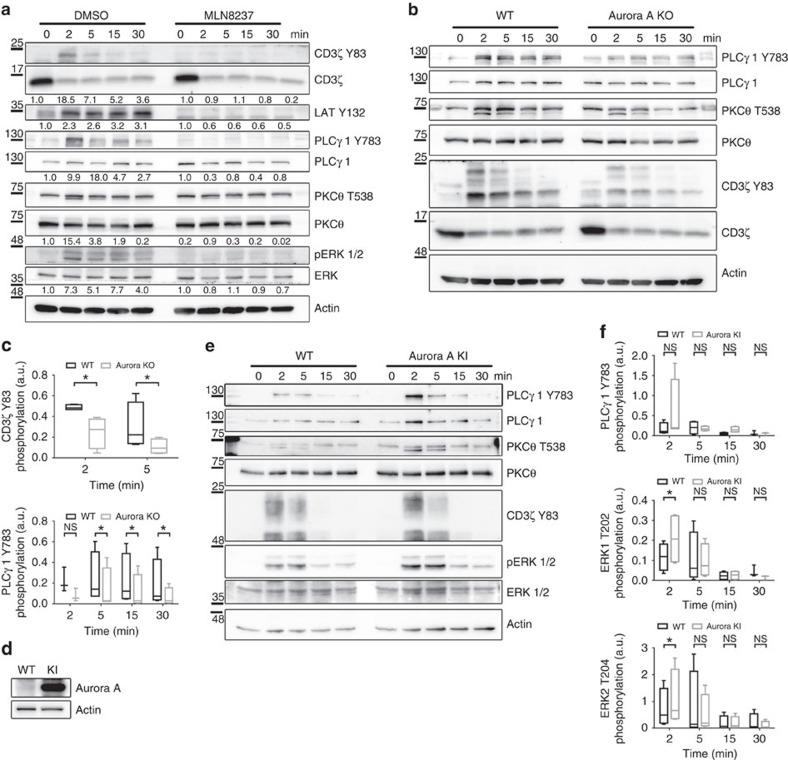
Aurora A gene ablation blocks TCR signalling pathways. (**a**) Immunoblottings showing phosphorylation of the indicated molecules in cell lysates of WT mouse CD4^+^ T cells pretreated with vehicle (DMSO) or Aurora A inhibitor (MLN8237) and activated for the indicated times with anti-CD3/CD28 antibodies. (**b**) Immunoblottings showing phosphorylation of the indicated molecules in cell lysates of Aurora KO and control CD4^+^ T cells conjugated for the indicated times with anti-CD3/CD28 antibodies. (**c**) Quantification of data from four independent experiments as in **b**. Error bars represent interquartile range. Medians were compared with a Friedman test (*P<0.05). n.s., nonsignificant. (**d**) Immunoblot analysis of Aurora A protein expression in CD4^+^ T cells isolated from *Col1a1tetO-Aurka/+*; *Rosa26rtTA/rtTA* mice and treated with doxycycline for 20 h to induce Aurora A expression (KI). Control cells (WT) were maintained without doxycycline. Actin is shown as a loading control. (**e**) Immunoblottings showing phosphorylation of the indicated molecules in cell lysates of Aurora KI CD4^+^ T cells conjugated for the indicated times with anti-CD3/CD28 antibodies. (**f**) Quantification of data from four independent experiments as in **e**. Error bars represent interquartile rage. Medians were compared with a Friedman test (*P-value<0.05). n.s., nonsignificant.

Pharmacologic inhibition of Aurora A also impaired early T-cell activation in mouse naive CD4^+^ T cells polyclonally stimulated with anti-CD3/CD28 (Fig. 8a). To further assess the function of Aurora A in primary naive T cells, we deleted Aurora A expression in CD4^+^ cells from the conditional Aurora KO mice and activated them with anti-CD3/CD28 antibodies. Tamoxifen-induced suppression of Aurora A expression in Aurka(lox/lox); RERT(ert/ert) cells (Fig. 3a) correlated with clear decreases in the phosphorylation of CD3ζ (Y83), LAT (Y132), PLCγ1 (Y783), PKCθ (T538) and ERK1/2 (T202/Y204) (Fig. 8b,c). CD4^+^ T cells from the conditional Aurora KO mice were also treated with MLN8237, obtaining a slight decrease in the phosphorylation of PLCγ1 (Y783) when compared with vehicle-treated CD4^+^ T cells from the conditional Aurora KO mice (Supplementary Fig. 5b). In complementary experiments, we examined a transgenic mouse model of Aurora A overexpression^33^. Naive CD4^+^ T cells isolated from lymph nodes and spleens of *Col1a1tetO-Aurka/+; Rosa26rtTA/rtTA* mice (Aurora KI) and controls were treated with doxycycline and IL-7 for 24 h, followed by activation with anti-CD3/CD28 antibodies. Doxycycline treatment increased Aurora A expression in the conditionally transgenic cells (Fig. 8d), correlating with increased levels of TCR-dependent signalling (Fig. 8e,f).

### Aurora A controls Lck kinase location and phosphorylation

**Figure 9:**
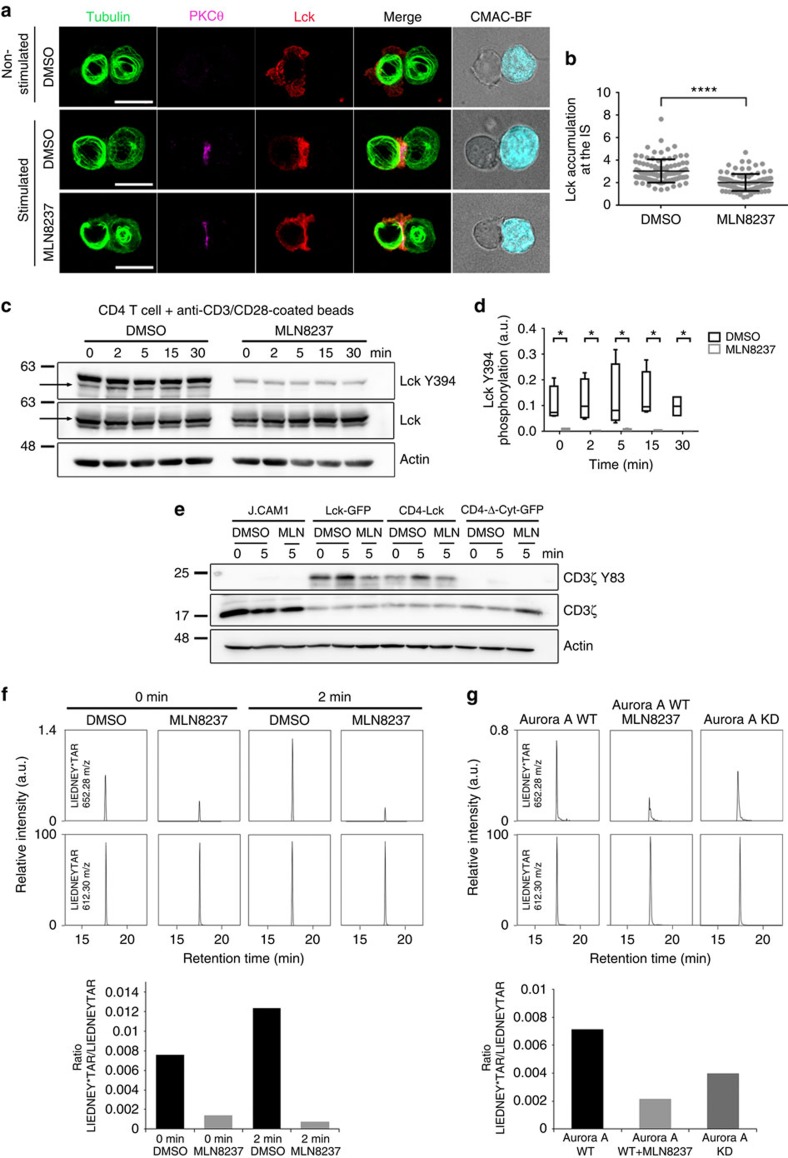
Aurora controls localization and phosphorylation of the tyrosine kinase Lck. (**a**) Maximum *Z* projection of *XYZ* stack of human Jurkat T cells pretreated with vehicle (DMSO) or Aurora A inhibitor (MLN8237) and conjugated with SEE-preloaded Raji B cells (APCs; 30 min). Cells were fixed and stained for α-tubulin–fluorescein isothiocyanate (FITC) (green), PKCθ (magenta) and Lck (red). Bright-field images are included. Scale bar, 10 μm. (**b**) Quantification of Lck accumulation at the IS in conjugates as in **a** from three independent experiments (DMSO, *n*=96. MLN8237, *n*=94). Data represent means±s.d. Means were compared with a *t*-test; ****P<0.0001. (**c**) Immunoblotting of Lck phosphorylation at Y394 in primary human CD4^+^ T cells. Cells were pretreated with DMSO or MLN8237 and conjugated for the indicated times with anti-CD3/CD28-coated beads. Total Lck and actin are included as loading controls. Arrows point Lck band. (**d**) Quantification of data from four independent experiments as in **c**. Error bars represent s.d. Medians were compared with a Friedman test (*P<0.05). (**e**) Immunoblots of CD3ζ phosphorylation in lysates of J.CAM1 T cells transfected with Lck-GFP, CD4-Lck or CD4-ΔCyt-GFP, pretreated with DMSO or MLN8237 and conjugated for 5 min with SEE-pulsed APCs. (**f**) T-cell lymphoblasts pretreated with DMSO or MLN8237 were activated or not with SEE-pulsed APCs (2 min) and subjected to IP using an anti-Lck antibody. The immunoprecipitates were subjected to MS analysis. Upper panel, MS/MS extracted ion chromatograms of the Y394-phosphorylated and non-modified forms of Lck peptide LIEDNEYTAR. Lower panel, phosphorylated:non-modified peak ratios. (**g**) Recombinant Lck was incubated with Aurora A WT (in the absence or presence of MLN8237) or Aurora A KD immunoprecipitated from nocodazole-treated (16 h), transfected HEK293 cells. Lck and Aurora were incubated for 30 min in the presence of ATP and the mixture analysed by MS. Upper panel, MS/MS extracted ion chromatograms of the Y394-phosphorylated and non-modified forms of Lck peptide LIEDNEYTAR. Lower panel, phosphorylated:non-modified peak ratios. See Supplementary Table 1 for representative MS/MS spectra of the phosphorylated and non-phosphoryated forms of the peptide at the peaks.

To study the mechanism underlying the earliest T-cell activation defects in the absence of Aurora A, we assessed the possible regulation of the Src kinase Lck by Aurora A. Lck phosphorylates CD3 ITAMs at tyrosine residues on TCR triggering and shows autophosphorylation activity towards its Y394 residue, an activatory residue^34^. By quantitative analysis of Lck accumulation at the IS we have detected a significant reduction in Lck relocation to the IS contact area, as a result of Aurora A inhibition in Jurkat T cells (Fig. 9a,b). In accordance with a perturbed Lck localization, pharmacologic inhibition of Aurora A in human primary CD4^+^ T cells impaired Lck autophosphorylation at Y394, a hallmark of its catalytic activity (Fig. 9c,d). Notably, these experiments showed that Lck-Y394 phosphorylation was impaired before TCR stimulation, suggesting a role of Aurora A in the maintenance of the preactivated pool of Lck^34^.

To analyse whether the effect of Aurora A on Lck activation is dependent on the intracellular traffic of Lck^27^ and taking into account that Lck recruitment at the IS is also driven by its association with CD4 (ref. 35), we decided to assess T-cell activation in a Lck-deficient cell line (J.CAM1 (refs 36, 37)) reconstituted with full-length Lck-GFP or murine CD4-Lck chimeric proteins. CD4-Lck is mainly localized at the plasma membrane^38^,^39^. A murine CD4 lacking its cytosolic tail and fused to GFP was used as a negative control^40^ (Fig. 9e; CD4-ΔCyt-GFP). We found that Lck-GFP expression rescued CD3 phosphorylation and thus T-cell activation in J.CAM1, whereas MLN8237 treatment prevented such an effect. Rescue of J.CAM1 signalling with CD4-Lck chimera was also prevented with the Aurora A inhibitor. Therefore, Aurora A activity is needed for Lck activity independently of its intracellular trafficking during IS formation.

Immunoprecipitation (IP) of Lck followed by mass spectrometry (MS) analysis revealed that Aurora A inhibition resulted in a decrease of Lck phosphorylation at the activation residue Y394 in resting and stimulatory conditions (Fig. 9f). This was further corroborated by *in vitro* kinase assays of purified recombinant Lck protein by immunoprecipitated Aurora A proteins. Although WT Aurora A protein keeps Lck phosphorylated at residue Y394, a KD form of Aurora is unable to maintain Lck phosphorylation at Y394 (Fig. 9g). Treatment with the Aurora A inhibitor corroborated the KD results (Fig. 9g). Together, these results highlight the relationship of Aurora A-mediated signal spreading at the IS with Lck location, phosphorylation and, therefore, regulation.

## Discussion

In this study we have analysed the influence of a well-known cell cycle regulator, Aurora A kinase, in T-cell activation. Our results provide novel evidence that Aurora A is a key regulator of early TCR-dependent signalling pathways and controls signalling vesicle and microtubular dynamics. However, the direct interaction of TCR/CD3 with Nck and actin polymerization at IS are not affected by Aurora A inhibition. Aurora A localizes at the IS and appears activated on antigen- and superantigen-driven T-cell activation. Early activation of Aurora A seems to be essential for TCR downstream signalling, leading to LAT and PLC activation. In addition, our data provide mechanistic insight into how Aurora A acts as master regulator of T-cell activation by controlling Lck phosphorylation and clustering at the IS.

Aurora A localization to centrosomes and along spindle MTs at the beginning of mitosis is well characterized^13^,^17^. The location of Aurora A in interphase is not well established, although the human protein atlas indicates that nuclear and cytoplasmic pools co-exist (http://www.proteinatlas.org/). Our data reveal that a fraction of active Aurora A (T288) appears at the IS contact area and a second pool is concentrated at the pericentrosomal area. The active form at the IS was observed on TCR stimulation, whereas the centrosome fraction seemed to be basally active in primary CD4^+^ T cells. The active pool and the total protein showed a similar pattern, indicating that there is an active redistribution of the protein on stimulation. This highlights the possibility that Aurora A autophosphorylation might have a role on its own localization at the IS, which is also supported by the fact that the expression of an Aurora A KD mutant provokes the delocalization of the active protein at the IS. However, further studies should be conducted to prove this view. The presence of two detectable pools suggest that Aurora A may play a possible dual role in controlling MT dynamics and T-cell activation. Although Aurora A can autophosphorylate, it is conceivable that other kinases are also involved in its activation. The MT-associated protein Tpx2 can activate Aurora A through its stabilization during cell division and prevents PP1 phosphatase from inactivating Aurora A^41^. Therefore, the distribution of activated Aurora A at the IS, a zone where a complex microtubular network is rapidly organized, may be responsible for its stabilization and activation, establishing a positive feedback for tubulin dynamics.

Aurora A contributes to centrosome maturation through the recruitment of MT nucleation factors. However, its absence does not prevent the formation of the centrosomal MT aster but instead affects the density of the aster formed in other systems^17^. Our TIRF microscopy analysis demonstrates that Aurora A controls growing MT arising from the MTOC on TCR activation, while having no apparent effect on MTOC translocation at the IS. In addition, during the M phase Aurora A is required for the recruitment of adaptor proteins such as NEDD1 for the correct formation of the mitotic spindle^42^. Previous work on proteins implicated in MT regulation such as EB1 or HDAC6 (refs 12, 43) showed a defect in late T-cell activation. The role of these proteins in MT cytoskeleton dynamics and T-cell activation seemed to be mainly related to the maintenance of the TCR signal rather than its initial activation. Aurora A might regulate late T-cell activation through a similar mechanism. Our data indicate that the decrease in the number of MTs nucleated near the contact area may affect polarized secretion from this area and vesicular trafficking at the IS and throughout the T cell. Hence, Aurora A inhibition prevents movement of CD3ζ vesicles around the MTOC almost completely, possibly reflecting a global effect on vesicle trafficking. In addition, as CD3 is tightly regulated by its cycle of degradation and recycling^44^, the absence of this pool of CD3ζ vesicles at the cSMAC may explain why the TCR signal cascade is not properly propagated. It has been proved that there is a pool of phosphorylated CD3ζ that, instead of going to a degradation pathway, keeps accumulated at the endosomal compartment, ready to maintain CD3ζ phosphorylation signalling^26^. Although Aurora A inhibition has no effect in TCR/CD3ɛ subunit surface clustering at the IS, the transport of vesicles of the CD3ζ subunit is clearly impaired. Taking into account the presence of this phosphorylated CD3ζ pool at the endosomal compartment, Aurora A might have an effect mainly over this recycling of the active CD3ζ and, therefore, over TCR signal propagation.

Although Aurora A contributes to actin cytoskeleton dynamics in mitosis and during mammary cell migration, no such effect was observed during IS formation by spreading T cells. Aurora-A-mediated phosphorylation of LIM kinase 1 at the centrosomes in prophase is essential for modulation of actin filaments and subsequent spindle formation. LIM kinase 1 acts by inactivating the phosphorylation of the actin depolymerizing family protein cofilin, thus stabilizing the cortical actin network during spindle orientation^45^. In mammary cell migration, Aurora A promotes increased expression of the cofilin phosphatase SSH1, resulting in cofilin activation and actin reorganization and migration^46^. However, our data show that Aurora A inhibition affects neither actin accumulation during IS formation nor cell spreading. Indeed, we found that Aurora-A-inhibited T cells form normal-shaped lamellae. During IS formation, Nck acts as a bridge between the TCR activation and actin cytoskeleton reorganization at the IS. When the TCR recognizes a specific antigen, a conformational change in the CD3ɛ chain unmasks a neoepitope to which Nck binds, leading to transmission of the activation signal through the actin cytoskeleton^28^. CD3ɛ-NcK association is not affected by Aurora A inhibition, a finding in accordance with the absence of changes in actin accumulation at the IS in MLN8237-pretreated T cells.

Our results show regulatory effects of Aurora A on early and late T-cell signalling. Inhibition of Aurora A abrogates proper T-cell activation determined by the phosphorylation profile of TCR signalling proteins such as CD3, and the adapter proteins and kinases LAT, PLCγ1 and PKCθ. These effects on TCR pathway phosphorylation events were observed in response to the Aurora A inhibitor MLN8237 and Aurora A gene ablation in mouse T cells, indicating that this is a specific consequence of Aurora A inhibition. The initial activation of T cells occurs at the plasma membrane; however, its continued progress requires the contribution of intracellular components such as the MTOC and the MT-dependent vesicular traffic and mitochondrial activity^3^. Thus, Aurora A contributes to the propagation of TCR activation to the intracellular compartment, leading to activation of genes such as *IL-2*, *CD69* and *CD25*. Moreover, the strength of T-cell activation can determine the ability of T cells to divide asymmetrically, thereby promoting functional differentiation into subpopulations of T cells that regulate the immune response^47^. Our data suggest that T lymphocytes defective in Aurora A do not become properly activated, possibly affecting the outcome of the adaptive immune response.

However, neither the defect on MT dynamics nor the impairment in CD3ζ vesicle transport can explain the blockade of the initial trigger of TCR signalling. These early defects of CD3ζ-dependent signalling in Aurora A-targeted cells are more likely to be explained by altered activity of Src kinases. This family includes Lck and Fyn, the first kinases to phosphorylate the ITAMs in CD3, which are required for full activation and signal transmission^5^,^48^. Our data demonstrate that Lck location and phosphorylation are altered by chemical inhibition of Aurora A, demonstrating that Aurora A controls TCR pathways dependent on CD3-ITAM phosphorylation. Nevertheless, the interaction of the kinase with the HSP90 and HSP70 chaperones is maintained in the presence of MLN8237, indicating that the inhibitor does not seem to affect its life time (Blas-Rus *et al*., unpublished data).

Previous work on Lck regulation has described the initial steps on the activation of this protein. A ‘standby' model has been proposed, where there is a pool of preactivated Lck whose phosphorylation does not change on TCR activation^34^. In this context, Lck function could be regulated through conformational changes, clustering and the spatio-temporal proximity to CD45 phosphatase, as well as with the exposition of the phosphorylatable ITAMs on TCR engagement^34^,^49^,^50^. However, other recent works detected a pool of Lck that became activated on TCR triggering assessed either by FRET-FLIM techniques^51^ or other different methods^52^. On the other hand, other studies addressed the importance of Lck spatial distribution in specific lipid rafts that rearrange on MHC–TCR binding^49^,^53^,^54^. In this regard, our results by complementary experimental strategies including western blot (WB) analysis of protein activation and MS analysis of endogenous Lck, and of *in vitro* kinase assays with purified recombinant Lck protein indicate that the activating Lck residue Y394 is phosphorylated in T cells before TCR stimulation. Remarkably, the targeting of Aurora A decreases Y394 phosphorylation and shows a delocalized Lck clustering at the IS. Reconstitution experiments in the Lck-deficient cell line J.CAM1 by either Lck-GFP or CD4-Lck, which retains Lck at the plasma membrane, revealed that Aurora A is required for TCR signalling in both situations. Taking into account the importance of Lck spatial distribution and proper phosphorylation for its activity, the dephosphorylation and mislocalization of Lck in the absence of Aurora A activity may explain the observed defects in TCR signalling pathways. A detailed analysis of other phosphorylated residues, including Ser/Thr, is needed to understand the complex regulation of Lck by Aurora A and this deserves future investigation. Furthermore, the assessment of how Aurora A controls Lck activity, either directly or indirectly through associated kinases, is an issue that remains to be explored.

In summary, our results show that Aurora A plays an important role in the early events initiated on TCR stimulation and unravel a novel molecular mechanism that regulates early signalling and cytoskeletal and vesicle dynamics in T cells. The prevention of T-cell activation by Aurora A inhibition has important clinical implications. Aurora A inhibitors are currently under evaluation for cancer therapy in Phase I–II clinical trials^55^. In these trials, aggressive B-cell and T-cell non-Hodgkin lymphomas have shown an overall positive response, promoting new Phase III studies. It will be important to define the extent to which the new function reported here participates in these responses and to determine whether the T-cell activation pathway can provide new biomarkers, critical for understanding these therapeutic effects. Very recently, a transcriptomic analysis points Aurora A as a targetable molecule for graft versus host disease prevention in a primate model^56^. Hence, our data provide a mechanistic explanation by how Aurora A controls T-cell activation. Given the importance of Aurora A inhibitors in cancer therapy, these results may provide new opportunities for treating lymphocyte diseases such as graft versus host disease, T-cell lymphomas or leukaemias.

## Methods

### Cells

The human Jurkat-derived T-cell lines J77 (Vαl.2 Vβ8+ TCR) and J.CAM1 (refs 36, 37), the lymphoblastoid B-cell lines Raji (Burkitt lymphoma; acquired from the DSMZ Organization; ACC-319) and Hom2 (HLA-DR1 EBV-transformed) were cultured in RPMI 1640+GlutaMAX–I+25 mM HEPES (Gibco–Invitrogen) supplemented with 10% fetal bovine serum (Hyclone, Thermofisher). The human Jurkat-derived CH7C17 cells (Vβ3+ transgenic TCR, specific for HA peptide) were grown in the same medium supplemented with 400 μg ml^−1^ hygromycin B (Roche Diagnostics) and 4 μg ml^−1^ puromycin (Invitrogen, Eugene, OR, USA). CH7C17 (ref. 57) clones expressing EB3-GFP were generated by CH7C17 transfection and post selection with G418 (1 mg ml^−1^). All lymphoid cell lines were tested for specific expression of CD (clusters of differentiation) with specific antibodies by flow cytometry. HEK293T cells were cultured in DMEM medium (Invitrogen) supplemented with 10% fetal bovine serum, 50 IU ml^−1^ penicillin and 50 μg ml^−1^ streptomycin, and exclusively used to produce and purify recombinant proteins. All cell lines were routinely tested for mycoplasm. Human peripheral blood mononuclear cells (PBMCs) were isolated from buffy coats obtained from healthy donors by separation on a Biocoll gradient (Biochrom) according to standard procedures. Monocytes were separated from PBMCs by a 30-min adherence step at 37 °C in RPMI supplemented with 10% FCS. Non-adherent cells were washed off and CD4^+^ T cells were purified from PBMCs using magnetic-activated cell sorting (MACS; Miltenyi Biotech). Non-adherent cells were obtained after 30 min of the adhesion step at 37 °C. To generate SEE-responsive human T lymphoblasts, PBMCs were cultured for 5 days in the presence of SEE (0.1 μg ml^−1^) and then phytohaemagglutinin (5 μg ml^−1^) was added for 2 days. To favour its proliferation, IL-2 (50 U ml^−1^) was added later to the medium every 2 days for a time period of 8 days. These studies were performed according to the principles of the Declaration of Helsinki and approved by the local Ethics Committee for Basic Research at the Hospital La Princesa (Madrid); informed consent was obtained from all human volunteers. These studies were performed according to the principles of the Declaration of Helsinki and approved by the local Ethics Committee for Basic Research at the Hospital La Princesa (Madrid); informed consent was obtained from all human volunteers.

### Mice

The Aurora A conditional model has been described^58^. These mice carry an *Aurka(lox)* conditional allele and the RERTert allele expressing an inducible Cre recombinase. After the appropriate crosses, we obtained the experimental *Aurka(lox/lox); RERT(ert/ert)* and control *Aurka(+/+); RERT(ert/ert)* mice used in this study. Cre activation on tamoxifen treatment induces conversion of the *Aurka(lox)* allele to the Aurka(Δ) allele. The Aurora kinase A (AurkA)-inducible mouse model has been reported recently^33^. This model was generated using the tetracycline-inducible single-copy transgenic system^59^ and carries the *M2-rtTA* gene inserted within the *Rosa26* allele and a cassette containing the Aurora-A complementary DNA under the control of the doxycycine-responsive promoter (tetO) inserted downstream of the *Col1a1* locus. The final mouse model, *Col1a1tetO-Aurka/+; Rosa26rtTA/rtTA*, overexpresses exogenous Aurora-A on doxycycline treatment in a wide range of proliferative and non-proliferative tissues and cells.

Both Aurora A mouse models were maintained in a mixed background (*129/Sv*, *CD1*, *C57BL/6J* and *FVB/N*). Mice were housed in the pathogen-free animal facility of the Centro Nacional de Investigaciones Oncológicas (Madrid) in accordance with the animal care standards of the institution. For experimentation, genotyped littermates, male or female mice of 7–9 weeks were used. These animals were observed on a daily basis and sick mice were killed humanely in accordance with the Guidelines for Humane Endpoints for Animals used in biomedical research. All animal protocols were approved by the Instituto de Salud Carlos III Committee for Animal Care and Research.

Mouse CD4^+^ T cells were obtained from single-cell suspensions of the spleen and mesenteric lymph node. The cell suspensions were incubated with biotinylated antibodies against CD8, CD16, CD19, CD24, CD117, MHC class II (I-Ab), CD11b, CD11c and DX5, and were subsequently incubated with streptavidin microbeads (MACS; Miltenyi Biotec). CD4^+^ T cells were negatively selected in an auto-MACS Pro Separator (Miltenyi Biotec). Cells were then labelled with antibodies to CD4 and CD25, and analysed by flow cytometry to confirm their purity and resting status. For conditional KO and knockin studies, mouse CD4^+^ T cells were cultured with tamoxifen for 96 h (Aurora A gene deletion model) or doxycycline for 20 h (Aurora A overexpression model) in RPMI 1640+GlutaMAX–I+25 mM HEPES (Gibco–Invitrogen) supplemented with 10% fetal bovine serum (Hyclone, Thermofisher), 50 IU ml^−1^ per ml penicillin, 50 μg ml^−1^ per ml streptomycin and 5 ng ml^−1^ per ml murine IL-7.

### Antibodies and reagents

The antibodies used in this study were anti-CD3ζ Y83 (ab68236; 1:1,000 for WB), anti-LAT Y132 (ab4476; 1:1,000 for WB), anti-Aurora A T288 (ab83968; 1:200 for immunofluorescence (IF)) and anti-Aurora A (a13824; [35C1]; 1:500 for WB) from Abcam; anti-α-Tubulin (T6199; clon DM1A; 1:2,000 for WB) and fluorescein isothiocyanate-conjugated anti-α-Tubulin (F2168; clon DM1A; 1:100 for IF) from Sigma; anti-ERK1/2 (SKU 13-6200; 1:500 for WB), anti-V5 (R960-25; 1:1,000 for WB and 0.5 μg per point for IP) and anti-Src pY418 (44-660G; that recognizes Lck Y394 (ref. 34), 1:1,000 for WB) from Invitrogen; anti-ERK1/2 T202/Y204 (44285; 1:1,000 for WB) from Calbiochem; anti-Aurora A (04-1037; 1:1,000 for WB) and anti-Lck (05-435; 1 μg per point for IP) from Millipore; anti-PKCθ (610090; 1:1,000 for WB), anti-Rac1 (610651; 1:1,000 for WB), anti-mouse CD3*ɛ* (553057; clon 2C11; 10 μg ml^−1^) and CD28 (553294; 5 μg ml^−1^) and anti-human CD28 (555725; 2 μg ml^−1^) from BD Pharmingen; anti-human CD3*ɛ* (317302; clon OKT3; 1:200 for IF) from BioLegend; anti-PKCθ T538 (9377S; 1:1,000 for WB), anti-PLCγ1 (2822S; 1:1,000 for WB), anti-PLCγ1 Y783 (#2821L; 1:1,000) and anti-Lck (2752; 1:1,000 for WB and 1:200 for IF) from Cell Signaling Tech; anti-PKCθ (sc-1875; 1:200 for IF) and anti-LAT (sc-7948; 1:500 for WB) from Santa Cruz. The anti-human CD3*ɛ* (300314; HIT3a; 5 μg ml^−1^) was from eBioscience. The anti-human-CD3ζ and anti-GST antibodies were produced in Dr B. Alarcón's laboratory (Centro de Biología Molecular Severo Ochoa, Madrid). Goat anti-Armenian hamster IgG was from Jackson ImmunoResearch (127-005-160; 10 μg ml^−1^). Cell tracker CMAC (7-amino-4-chloromethylcoumarin; C2110, 0.1 μM) was from Molecular Probes–Invitrogen. Enterotoxins E (SEE; 0.3 μg ml^−1^) and B (SEB; 5 μg ml^−1^) from *Staphylococcus aureus* were purchased from Toxin Technologies; the HA peptide (200 μg ml^−1^) was synthesized by Lifetein LLC. Recombinant human Lck, histidine tagged was from MBL (RB-P3043). The Aurora A inhibitor MLN8237 and Aurora B inhibitor AZD1152 were from Selleckchem. Prolong Gold anti-fade mounting medium (P-36934), phalloidin conjugated to Alexa Flour 647 (A-22287; 1:100 for IF), goat anti-rabbit and goat anti-mouse highly cross-adsorbed secondary antibodies conjugated to Alexa Fluor 488 (A-11034 and A-11029, respectively; 1:500 for IF), 568 (A11036 and A-11031, respectively; 1:500 for IF) or 647 (A-21443 and A-21236, respectively; 1:500 for IF), donkey anti-goat highly cross-adsorbed secondary antibody conjugated to Alexa Fluor 647 (A-21447; 1:500 for IF) and donkey anti-rabbit secondary antibody conjugated to Alexa Flour 555 (A-31572; 1:500 for IF) were from Thermofisher Scientific. Fibronectin and Poly-L-Lys were from Sigma. Horseradish peroxidase-conjugated secondary antibodies for WB (anti-rabbit 31460, mouse 31430 or goat IgG+IgM 31460) were from Pierce–Thermofisher Scientific. Murine IL-7 was from PreproTech (217-17).

### Plasmids and siRNAs and transfection

The plasmid encoding GFP-EB3 was generously provided by Dr A. Akhmanova (Utrecht University, Utrecht, The Netherlands)^24^. The plasmids encoding WT or KD Aurora A-GFP and WT or KD V5-Aurora A were reported previously^60^. CD4-Lck^38^ in pRC3.1 plasmid was sub-cloned in the laboratory of Dr M Alonso; Dr M Alonso also provided the Lck-GFP^39^ and CD4ΔCyt-GFP^40^ constructs (CBM, Madrid, Spain), and Actin–mCherry-expressing CH7C17 T-cell clones were generated in the laboratory of Dr JM Serrador (CBM). The GST-PAK-CD (p21-activated kinase-CRIB-Domain)^30^ was generously provided by Dr Collard (NKI, Amsterdam, The Netherlands). The plasmid pGEX2TK (Pharmacia) was used as control. T-cell lines were transfected with specific double-stranded siRNA against human Aurora Kinase A 3′-untranslated region (5′-CCCUCAAUCUAGAACGCUA-3′)^61^ or a scramble negative control (5′-CUAGGGUGCCGAGUGUGUU-3′). For transfection, T-cell lines were centrifuged at 1,200 r.p.m. for 5 min and washed with Hank's balance salt solution(HBSS); Lonza) and resuspended in Opti-Mem I (Gibco–Invitrogen) (15 × 10^6^ cells in 400 μl). Corresponding plasmids (10 μg) were added to cell lines and transfection was performed with the gene-pulser III system from Bio-Rad Laboratories (240 V, 975 mΩ, ∼27 ms). After electroporation, cells were cultured in 9 ml RPMI 1640+GlutaMAXTM–I+25 mM HEPES medium. After 4 h, 500 μl fetal bovine serum was added to the cell medium. Experiments were performed 24 h after transfection. For mouse and human primary CD4^+^ T cells, corresponding plasmids (10 μg) were added to cells and transfection was performed with the Nucleofector I from Amaxa Biosystems (X-01). The plasmids encoding Aurora A-V5 WT or KD (24 μg) were tranfected with Lipofectamine (Invitrogen) in HEK293T cells. Experiments were performed 24 h after transfection.

### T cell activation and lysis for pull-down and immunoblotting

For human TCR stimulation, T cells were incubated for the indicated times with latex microbeads (6.4 μm diameter) conjugated to anti-CD3 antibody (10 μg ml^−1^) and anti-CD28 antibody (5 μg ml^−1^). For mouse TCR stimulation, T cells were incubated with anti-CD3 antibody (10 μg ml^−1^) and anti-CD28 antibody (5 μg ml^−1^) for 15 (4 °C) followed by incubation with goat anti-Armenian hamster IgG for 15 min (4 °C). For antigen stimulation, Raji cells were pulsed with 0.3 μg ml^−1^ SEE (30 min) and mixed with J77 or J.CAM1 cells (1:5); alternatively, Hom2 cells pulsed with 200 μg ml^−1^ HA peptide (2 h) or with 5 μg ml^−1^ SEB (30 min) and were mixed with CH7C17 cells (1:5) in HBSS. Where indicated, cells were pretreated with MLN8237 (10 μM) or AZD1152 (100 nM), or vehicle for 45 min at 37 °C in HBSS before stimulation with the corresponding APC or anti-CD3 and anti-CD28 antibodies. Cells were centrifuged at low speed for the indicated times at 37 °C to favour the formation of conjugates. Cells were lysed in 5 mM Tris-HCl pH 7.5 containing 1% NP40, 0.2% Triton X-100, 150 mM NaCl, 2 mM EDTA, 1.5 mM MgCl_2_, and phosphatase and protease inhibitors. Lysates were spin at 14,000 r.p.m. (4 °C, 10 min) to remove debris and nuclei. For GST-Nck or GST-PAK-CD, pull-down assay experiments were performed as described previously^28^,^30^. Proteins were resolved by SDS–PAGE and transferred to nitrocellulose membranes. After blocking with TBS containing 0.2% TWEEN and 5% BSA, membranes were blotted with primary antibodies (o/n at 4 °C) and peroxidase-labelled secondary antibodies (30 min), and detected with the ImageQuant LAS-4000 chemiluminiscence and fluorescence imaging system (Fujifilm). Source images from relevant WB are available in the Supplementary Figs 6–9.

### Cell conjugate and IF and IS analysis

Raji B cells or Hom2 B cells were washed once with HBSS and loaded with the CMAC cell tracker (10 μM) and with SEE or SEB for 30 min or HA peptide for 2 h at 37  °C. T cells (1 × 10^5^ cells) were mixed with the corresponding APC (1:1) and plated onto Poly-L-Lys-coated slides (50 μg ml^−1^; 1 h at 37 °C). Cells were allowed to settle for 20 min at 37 °C, fixed with 4% paraformaldehyde and 0.12 mM sucrose in PHEM (60 mM PIPES, 25 mM Hepes, 5 mM EGTA and 2 mM MgCl_2_), and permeabilized for 5 min at room temperature with 0.2% Triton X-100 in immunofluorescence solution (PHEM containing 3% BSA, 100 μg ml^−1^ γ-globulin and 0.2% azide). Cells were blocked for 30 min with immunofluorescence solution and stained with the indicated primary antibodies (5 μg ml^−1^) followed by Alexa Fluor 488-, 568- or 647-labelled secondary antibodies, Alexa-conjugated phalloidin (5 μg ml^−1^) or fluorescein isothiocyanate-conjugated anti-α-tubulin (0.1 μg ml^−1^). Cells were mounted on Prolong Gold and analysed with a Leica SP5 confocal microscope (Leica) fitted with a HCX PL APO × 63/1.40–0.6 oil objective and images were processed and assembled using Image J software (http://rsbweb.nih.gov/ij/) and Photoshop software. For quantification in individual ISs, we used a home-made plugin for Image J software (http://rsbweb.nih.gov/ij/) called ‘*Synapse Measures*'. By comparing fluorescence signals from multiple regions of the T cell, APC, IS and background fluorescence, the programme yields accurate measurements of localized immunofluorescence. A detailed description of *Synapse Measures* including the algorithms used is described^62^.

### IP and MS and phosphorylation

For Lck IP assay, human lymphoblast pretreated with MLN8237 (10 μM) or vehicle (DMSO) for 30 min were activated with Raji preloaded with SEE for 2 min at 37 °C. Next, cells were lysated for 40 min at 4 °C in extraction buffer with 5 mM Tris-HCl pH 7.5 containing 0.5% NP40, 150 mM NaCl, 2 mM EDTA, 1.5 mM MgCl2, and phosphatase and protease inhibitors. Lysates were spun at 14,000 r.p.m. (4 °C, 10 min) to remove debris and nuclei. The anti-Lck antibody was allowed to bind with Protein G-conjugated sepharose beads (GE Healthcare) overnight at 4 °C and then mixed with the extracts. The mixture was left in agitation at 4 °C for 2 h and then beads were washed ten times with the same buffer used for lysate without detergents. As a control, we used beads preincubated with the extracts. For Aurora A IP, V5-Aurora A WT- or KD-transfected HEK293T cells were lysated with RIPA buffer, with 1% Triton X-100, 0.5% deoxycholate (Sigma-Aldrich), 0.1% SDS in Tris buffer saline and sonicated (3 × 30 s pulses). The anti-V5 antibody was mixed with the extracts and left in agitation at 4 °C for 2 h, and then Protein G-conjugated sepharose (GE Healtcare) was added for 1 h in agitation at 4 °C. Beads were washed three times with the buffer kinase with 20 mM Hepes pH 7.4 containing 150 mM KCl, 10 mM MgCl_2_, 1 mM EGTA, 0.5 mM dithiothreitol, and phosphatase and protease inhibitors, and once with buffer kinase plus NaCl 0.5 mM. Beads were incubated with 0.5 μg of recombinant Lck in buffer kinase plus 10 mM ATP during 30 min at 30 °C. For proteomic analysis, the samples were trypsin-digested using the whole proteome in-gel digestion protocol^63^. The peptides produced by digestion were vacuum dried and redissolved in 1% trifluoroacetic acid for desalting in reversed-phase C-18 extraction cartridges (Oasis, Waters Corporation, Milford, MA, USA). High-resolution parallel reaction monitoring of phosphorylated peptides was carried out on an Easy nLC 1000 nano-HPLC apparatus (Thermo Scientific, San Jose, CA, USA) coupled to a hybrid linear ion trap-orbitrap (Orbitrap Elite, Thermo Scientific). Peptides were suspended in 0.1% formic acid and then loaded onto a C-18 reversed-phase nano-column (75 μm I.D., 50 cm) and separated in a continuous gradient consisting of 8–30% B for 15 min and 30–90% B for 2 min (B=90% acetonitrile, 0.1% formic acid) at 200 nl min^−1^. Peptides were ionized using a Picotip emitter nanospray needle (New Objective, Woburn, MA, USA). Each MS run consisted of enhanced FT-resolution spectra (30,000 resolution) in the 390–1,600 *m*/*z* range followed by data-independent MS/MS spectra of 11 parent ions acquired along the chromatographic run. The AGC target value in the Orbitrap for the survey scan was set to 1,000,000. Fragmentation in the linear ion trap was performed at 35% normalized collision energy with a target value of 10,000 ions and the dynamic exclusion was set to 0.5 min. Data analysis was performed with Xcalibur 2.2 (Thermo Scientific).

### Time-lapse confocal and TIRF movie microscopy

For cell conjugates, 3D imaging was performed with CMAC-loaded Raji APCs (5 × 10^5^; SEE-pulsed (Jurkat cells), SEB-pulsed (CH7C17 cells) or unpulsed) and were allowed to adhere to fibronectin-coated coverslips in Attofluor open chambers (Molecular Probes–Invitrogen) at 37 °C in a 5% CO_2_ atmosphere or in glass-bottom dishes (No. 1.5 Mattek; Ashland, MA, USA). The cells were maintained in 1 ml HBSS (1% fetal bovine serum and 25 mM HEPES). Cells were pretreated with the MLN8237 inhibitor and maintained in its presence during imaging when needed. T cells were added (1:1 ratio) and a series of fluorescence and differential interference contrast or bright-field frames were captured using a TCS SP5 confocal laser scanning unit attached to an inverted epifluorescence microscope (DMI6000) fitted with an HCX PL APO × 63/1.40–0.6 oil objective. Images were acquired and processed with the accompanying confocal software (LCS; Leica) and Image J software (http://rsbweb.nih.gov/ij/). For 3D imaging of MT growing, cells were allowed to settle onto glass-bottom dishes coated with anti-CD3 (10 μg ml^−1^) and anti-CD28 (3 μg ml^−1^) monoclonal antibodies specific for human or mouse T cells and XYZ series were captured with the resonant scanner of the TCS SP5 confocal (8,000 Hz) each 1.2 s or 1.1 s. Cells were pretreated with the MLN8237 inhibitor and maintained in its presence during imaging. For TIRF microscopy, T cells stably expressing EB3-GFP or transfected with EB3-GFP and CD3ξ-mCherry were allowed to settle onto glass-bottom dishes coated with anti-CD3 (10 μg ml^−1^) and anti-CD28 (3 μg ml^−1^). Cells were pretreated with the MLN8237 inhibitor and it was maintained in the imaging medium during acquisition. Recording was initiated 3 min after cells were plated and cells were visualized with a Leica AM TIRF MC M system mounted on a Leica DMI 6000B microscope coupled to an Andor-DU8285_VP-4094 camera fitted with a HCX PL APO × 100.0, 1.46 oil objective. For mCherry–β-actin-expressing T cells, recording was initiated on addition of cells to the glass-bottom dishes. Images were processed with the accompanying confocal software (LCS; Leica). The laser penetrance used was 150 or 200 nm for both laser channels (488 and 561 nm), using the same objective angle. Time-lapse settings were optimized for each type of experiment and are specified throughout the text. Synchronization was performed with the accompanying Leica software and images were processed with Leica software, Matlab and Image J software (http://rsbweb.nih.gov/ij/).

### Quantitative real-time PCR

Reverse transcriptase–PCR was performed with 1 μg of RNA isolated with Trizol RNA reagent (Invitrogen) from CD4^+^ T cells obtained from healthy donors. mRNA levels of IL-2, CD25 and CD69 were determined in triplicate using the Power SYBR Green PCR master mix obtained from Applied Biosystems (Warrington, UK). Expression levels were normalized to the expression of glyceraldehyde-3-phosphate dehydrogenase. Primer sequences are listed in Supplementary Table 2.

## Additional information

**How to cite this article**: Blas-Rus, N. *et al*. Aurora A drives early signalling and vesicle dynamics during T-cell activation. *Nat. Commun.* 7:11389 doi: 10.1038/ncomms11389 (2016).

## Supplementary Material

Supplementary Information Supplementary Figures 1-9 and Supplementary Tables 1-2

Supplementary Movie 1 4D imaging of Microtubule growing in control and Aurora A-inhibited Jurkat cells. Control Jurkat T cells transfected with EB3-GFP were pre-treated with MLN8237 or vehicle, allowed to settle on stimulatory anti-CD3/CD28-coated surfaces and recorded under a confocal microscope. Fluorescence and bright field images for XYZ-stacks were taken every 1.2 s. MLN8237 or vehicle was present in the imaging medium. Movie was mounted at 10 fps.

Supplementary Movie 2 4D imaging of Microtubule growing in control and Aurora A-inhibited WT and KO cells. WT and KO cells were transfected with EB3-GFP, pre-treated with MLN8237 or vehicle and allowed to settle on stimulatory anti-CD3/CD28-coated surfaces and recorded under a confocal microscope. Fluorescence and bright field images for XYZ-stacks were taken every 1.2 s. MLN8237 or vehicle was present in the imaging medium. Movie was mounted at 10 fps.

Supplementary Movie 3 Tracking of EB3-GFP-decorated, growing TIPs at the IS in control Jurkat cells. Control Jurkat T cells stably expressing EB3-GFP were allowed to settle on stimulatory anti-CD3/CD28-coated surfaces and recorded under a TIRFm, at a 150 nm of penetrance upon excitation with a 488 nm laser. Images were taken every 300 ms. MLN8237 or vehicle was present in the imaging medium. Imaris Software was used to recognize fluorescence corresponding to the decorated tips and to calculate the trajectories and growing speed of the tips. Movie was mounted at 30 fps.

Supplementary Movie 4 Tracking of EB3-GFP-decorated, growing TIPs at the IS in Aurora A-inhibited Jurkat cells. MLN8237-treated Jurkat T cells stably expressing EB3-GFP were allowed to settle on stimulatory anti-CD3/CD28-coated surfaces and recorded under a TIRFm, at a 150 nm of penetrance upon excitation with a 488 nm laser. Images were taken every 300 ms. MLN8237 or vehicle was present in the imaging medium. Imaris Software was used to recognize fluorescence corresponding to the decorated tips and to calculate the trajectories and growing speed of the tips. Movie was mounted at 30 fps.

Supplementary Movie 5 Tracking of EB3-GFP-decorated, growing TIPs at the IS in control CD4^+^ T cells. CD4^+^ T cells isolated from Aurka(lox/lox); RERT(ert/ert) and transfected with EB3-GFP were allowed to settle on stimulatory anti-CD3/CD28-coated surfaces and recorded under a TIRFm, at a 150 nm of penetrance upon excitation with a 488 nm laser. Images were taken every 300 ms. MLN8237 or vehicle was present in the imaging medium. Imaris Software was used to recognize fluorescence corresponding to the decorated tips and to calculate the trajectories and growing speed of the tips. Movie was mounted at 30 fps.

Supplementary Movie 6 Tracking of EB3-GFP-decorated, growing TIPs at the IS in Aurora A-deficient CD4^+^ T cells. CD4^+^ T cells isolated from Aurka(lox/lox); RERT(ert/ert) treated with tamoxifen and transfected with EB3-GFP were allowed to settle on stimulatory anti-CD3/CD28-coated surfaces and recorded under a TIRFm, at a 150 nm of penetrance upon excitation with a 488 nm laser. Images were taken every 300 ms. MLN8237 or vehicle was present in the imaging medium. Imaris Software was used to recognize fluorescence corresponding to the decorated tips and to calculate the trajectories and growing speed of the tips. Movie was mounted at 30 fps.

Supplementary Movie 7 Tracking of CD3ζ-bearing vesicles at the IS in control Jurkat cells. Control Jurkat T cells transfected with CD3ζ-mCherry were allowed to settle on stimulatory anti-CD3/CD28-coated surfaces and recorded under a TIRFm, at a 200 nm of penetrance upon excitation with a 561 nm laser. Images were taken every 100 ms. MLN8237 or vehicle was present in the imaging medium. Imaris Software was used to recognize fluorescence corresponding to the vesicles and to calculate the trajectories, their duration and the speed of the vesicles. Movie was mounted at 20 fps.

Supplementary Movie 8 Tracking of CD3ζ-bearing vesicles at the IS in Aurora A-inhibited Jurkat cells. MLN8237-treated Jurkat T cells transfected with CD3ζ-mCherry were allowed to settle on stimulatory anti-CD3/CD28-coated surfaces and recorded under a TIRFm, at a 200 nm of penetrance upon excitation with a 561 nm laser. Images were taken every 100 ms. MLN8237 or vehicle was present in the imaging medium. Imaris Software was used to recognize fluorescence corresponding to the vesicles and to calculate the trajectories, their duration and the speed of the vesicles. Movie was mounted at 20 fps.

Supplementary Movie 9 Tracking of CD3ζ-bearing vesicles at the IS in control or Aurora A inhibited CD4^+^ T cells. CD4^+^. T cells isolated from Aurka(lox/lox); RERT(ert/ert), transfected with EB3-GFP and CD3ζ-mCherry, treated with MLN8237 or vehicle and allowed to settle on stimulatory anti-CD3/CD28-coated surfaces. Recording was performed under a TIRFm, at a 200 nm of penetrance upon excitation with a 561 nm laser. Images were taken every 110 ms. MLN8237 or vehicle was present in the imaging medium. Movie was mounted at 20 fps.

Supplementary Movie 10 4D imaging of Actin ring formation in conjugates of in control and Aurora A-inhibited Jurkat cells. Control Jurkat T cells transfected with mCherry-β-actin were pre-treated with MLN8237 or vehicle, allowed to settle on stimulatory anti-CD3/CD28-coated surfaces and recorded under a confocal microscope. Fluorescence and bright field images for XYZ-stacks were taken every 25 s. MLN8237 or vehicle was present in the imaging medium. Bar, 10 μm. Movie was mounted at 10 fps.
