# Supplementary material for: Aurora A drives early signalling and vesicle dynamics during T-cell activation
Source: Nat Commun. 2016 Apr 19;7:11389. doi: 10.1038/ncomms11389 (PMC4838898; doi:10.1038/ncomms11389)
Supplement: Supplementary Information — Supplementary Figures 1-9 and Supplementary Tables 1-2 [file ncomms11389-s1.pdf]

Supplementary Figure 1

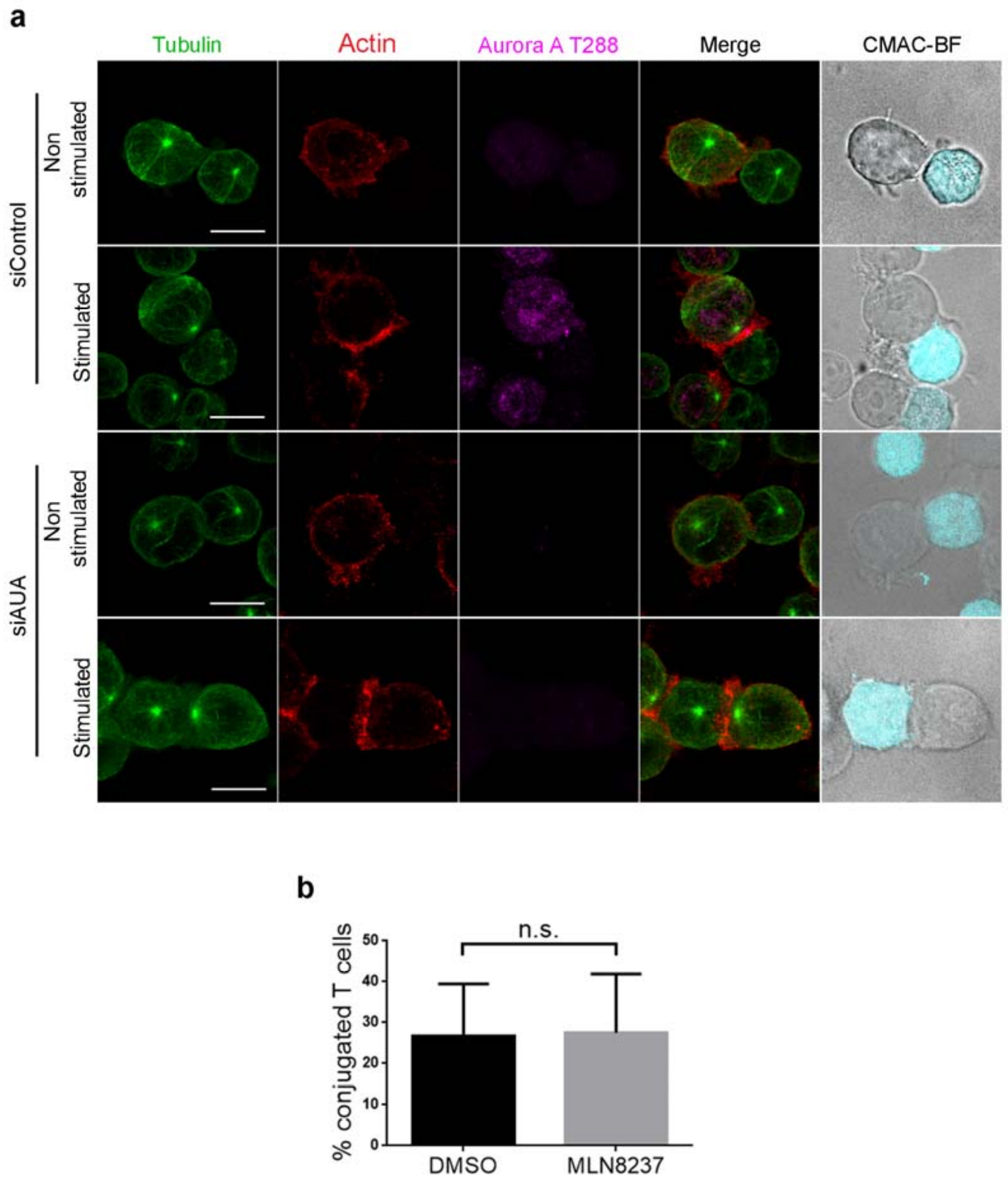

Supplementary Figure 1. Specific silencing of Aurora A. **(a)** Maximum intensity Z-projection of a confocal XYZ-stack of Jurkat T cells transfected with a specific siRNA against Aurora A (siAUA) or a scrambled negative control (siControl) and conjugated with SEE-pulsed Raji B cells. Cells were incubated for 30 min, fixed and stained for α-tubulin (green), T288-phosphorylated Aurora A (magenta) and actin (red). A merged fluorescence image is shown. The right-hand image shows Raji cells labeled with CMAC cell tracker (cyan) and bright field. Bar, 10 µm. **(b)** Quantification of the percentage of conjugates formed between J77 T cells, pre-treated with DMSO or MLN8237, and SEE-pulsed Raji B cells (ratio 1:1).

Supplementary Figure 2

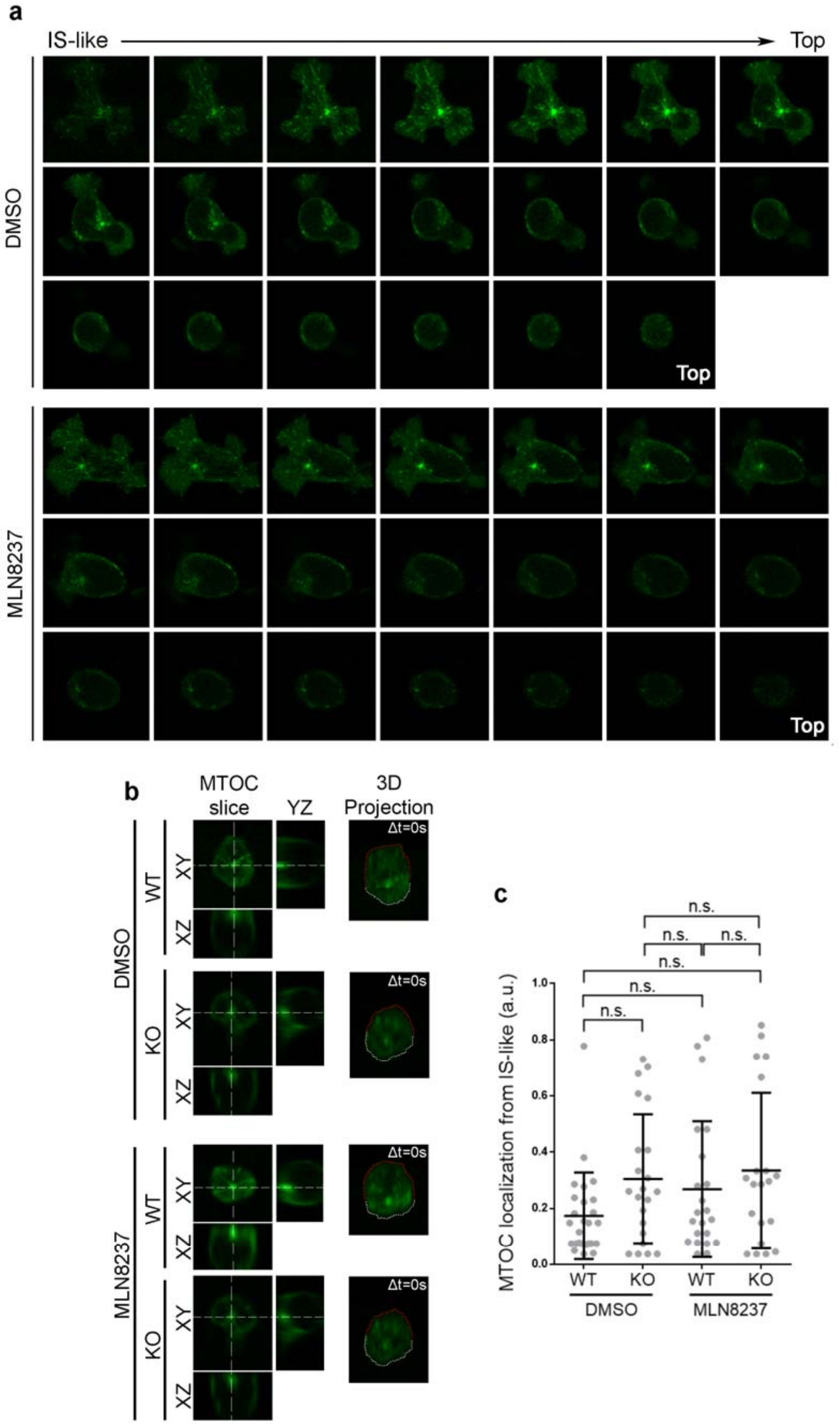

Supplementary Figure 2. Imaging of EB3-GFP decorated TIPs. (**a**) Single slices from XYZ-stacks of 4D imaging of EB3-GFP-transfected human CH7C17 T cells treated with DMSO or MLN8237 and platted onto anti-CD3/CD28 stimulating antibodies (time 0s). (**b**) Orthogonal and 3D projections from XYZ-stacks of WT and KO mouse cells, pre-treated with DMSO or MLN8237 (time 0s). Dotted white and red lines indicate contact with substrate and media in the 3D projection, respectively. **(c)** Ratio of the MTOC location from the IS-like (n=26 in WT, 22 in KO and WT+MLN8237 and n=20 in KO+MLN8237).

Supplementary Figure 3

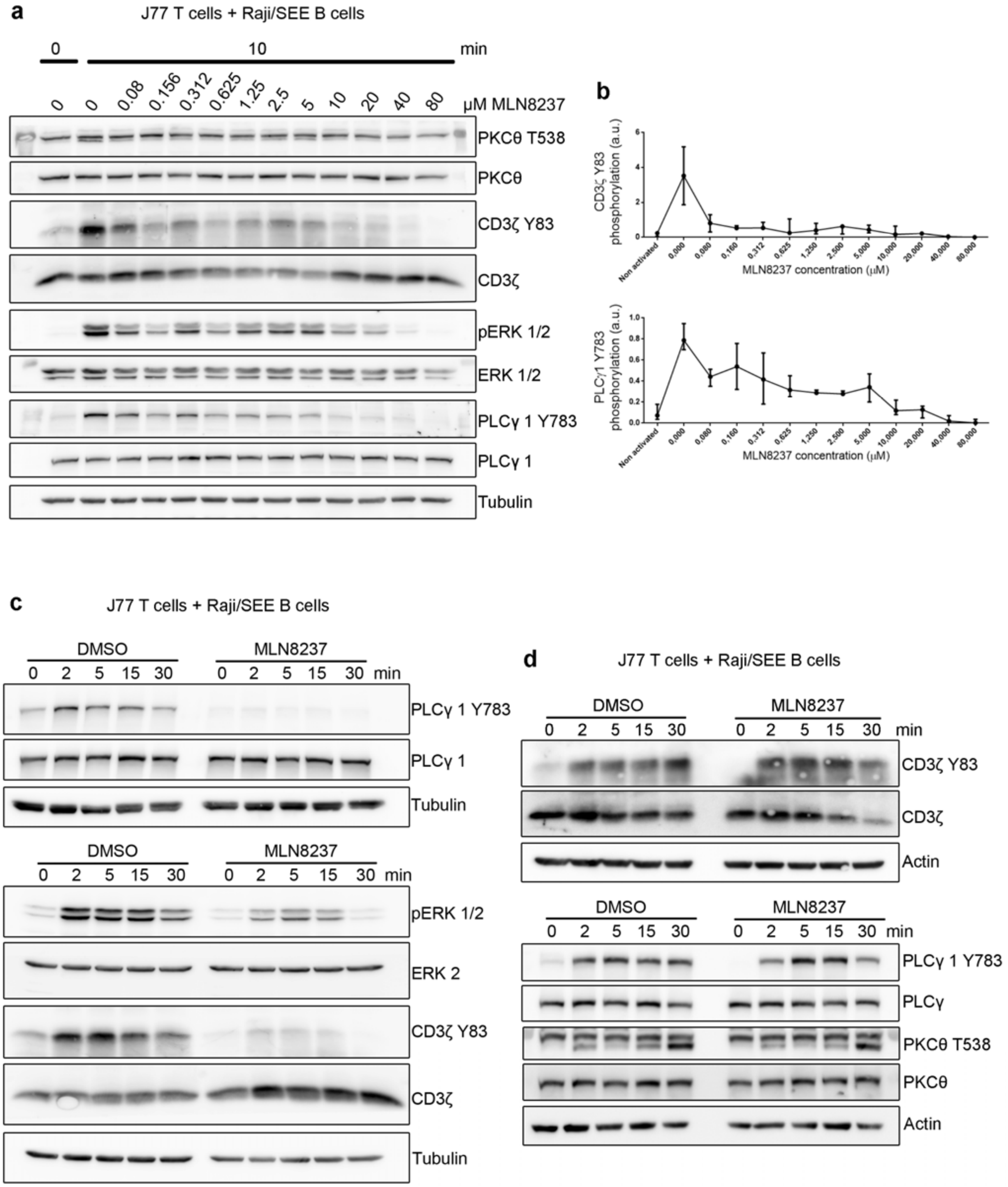

Supplementary Figure 3. Dose-response effect of the Aurora A inhibitor MLN8237 on T cell activation. (a) Immunoblots showing phosphorylation of the indicated molecules in lysates of J77 Jurkat T cells pretreated with vehicle (DMSO) or Aurora A inhibitor (MLN8237) at the concentrations indicated and conjugated for 10 minutes with SEE-pulsed Raji B cells. **(b)** Quantification of blots as in **a** from three independent experiments. Medians are shown. Error bars represent interquartile range. (**c**) Immunoblots showing phosphorylation of the indicated molecules in lysates of J77 Jurkat T cells conjugated for the indicated times with SEE-pulsed Raji B cells in presence of DMSO or MLN8237. **(d)** Immunoblots showing phosphorylation of the indicated molecules in lysates of J77 Jurkat T cells pretreated with DMSO or MLN8237 for 30 min, extensively washed and conjugated for the indicated times with SEE-pulsed Raji B cells.

Supplementary Figure 4

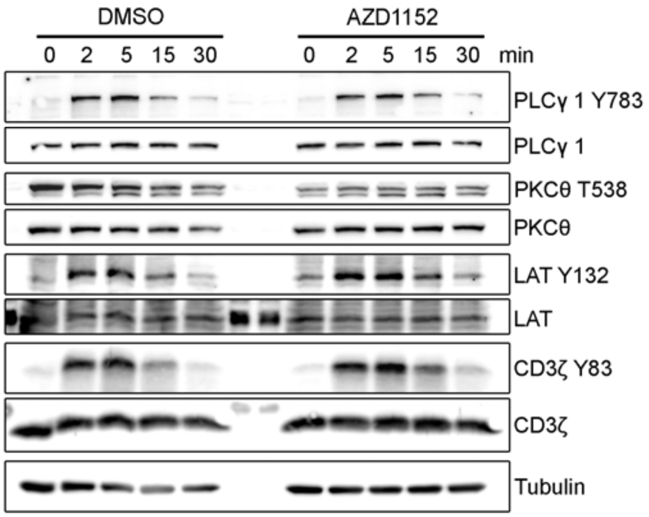

Supplementary Figure 4. TCR signaling is not affected by Aurora B inhibition. Immunoblots showing phosphorylation of the indicated molecules in lysates of J77 Jurkat T cells pretreated with vehicle (DMSO) or Aurora B inhibitor (AZD1152, 100 nM) and conjugated for the indicated times with SEE-pulsed Raji B cells.

Supplementary Figure 5

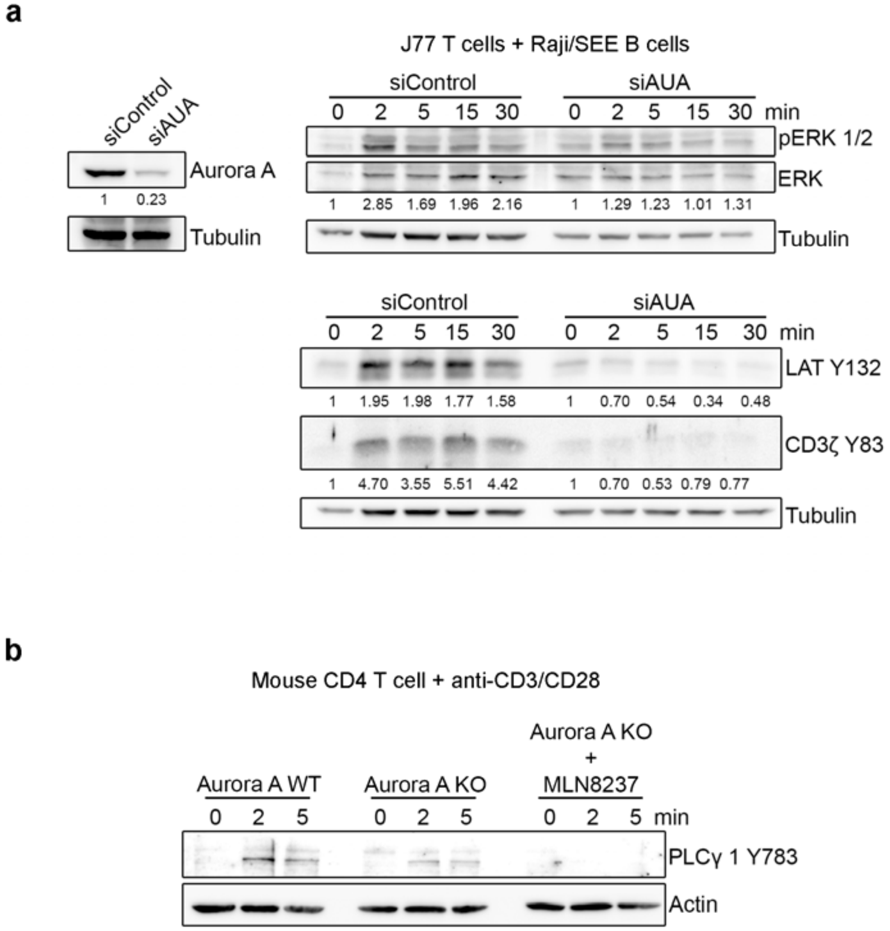

Supplementary Figure 5. TCR signaling is impaired by Aurora A silencing. (a) Immunoblots showing phosphorylation of the indicated molecules in lysates of J77 Jurkat T cells transfected with a specific siRNA against Aurora A (siAUA) or a scrambled negative control (siControl) and conjugated for the indicated times with SEE-pulsed Raji B cells**. (b)** Immunoblots of PLCγ1 phosphorylation (pY783) in lysates of Aurora KO pretreated with vehicle (DMSO) or the Aurora A inhibitor (MLN8237) and control mouse $CD4^+$ T cells conjugated for the indicated times with anti-CD3/CD28 antibodies.

Supplementary Figure 6

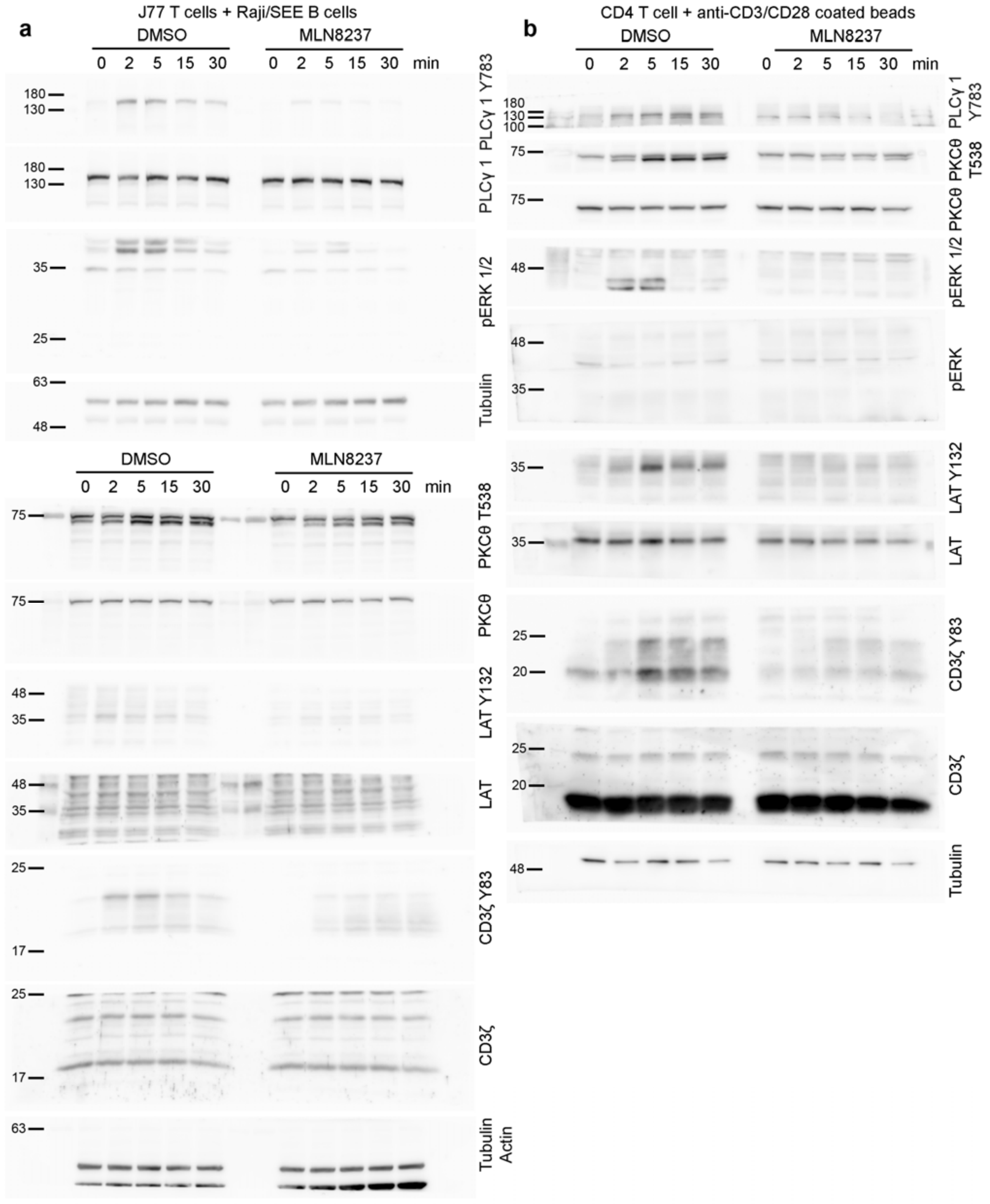

Supplementary Figure 6. Uncropped Western blots. (a) Western blots correspond to Fig. 6a. (**b**) Western blots correspond to Fig. 6c. Molecular weights are indicated.

Suplementary Figure 7

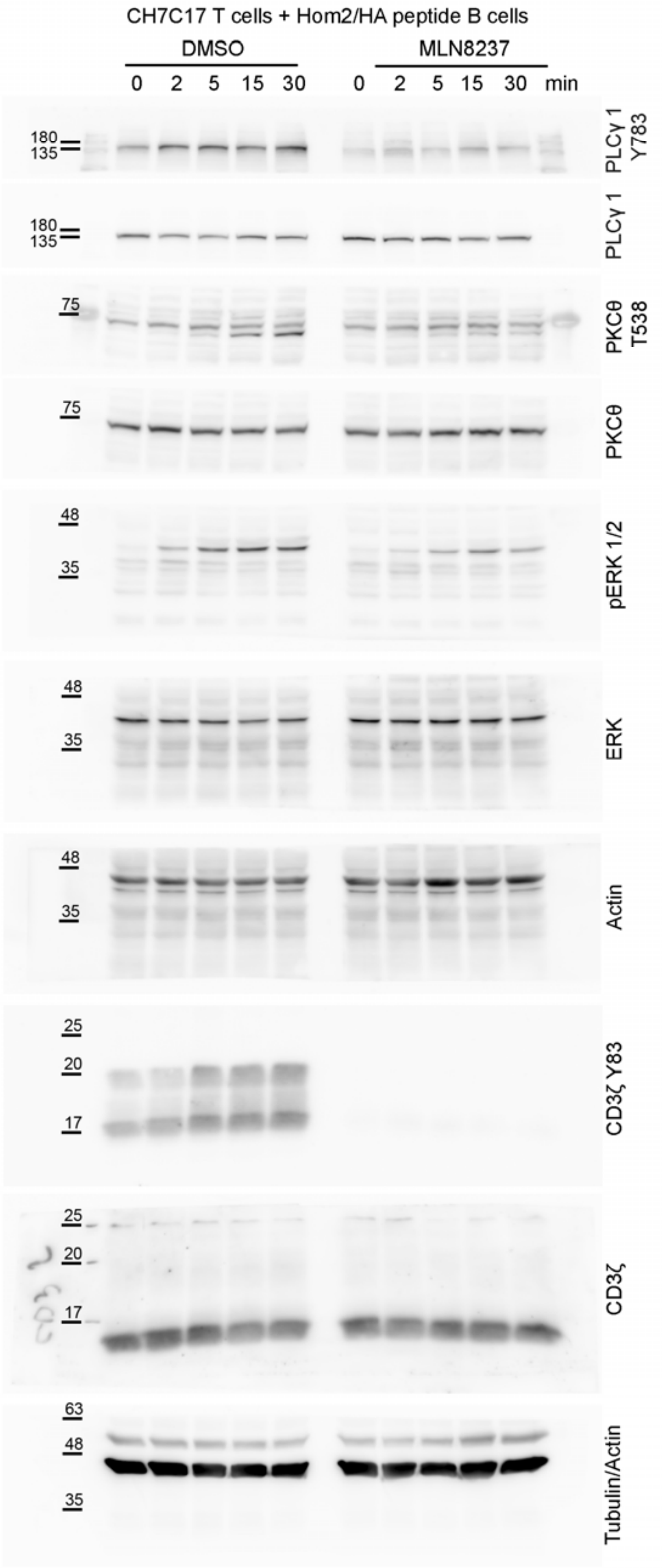

Supplementary Figure 7. Uncropped Western blots. Western blots correspond to Fig. 7a. Molecular weights are indicated.

Suplementary Figure 8

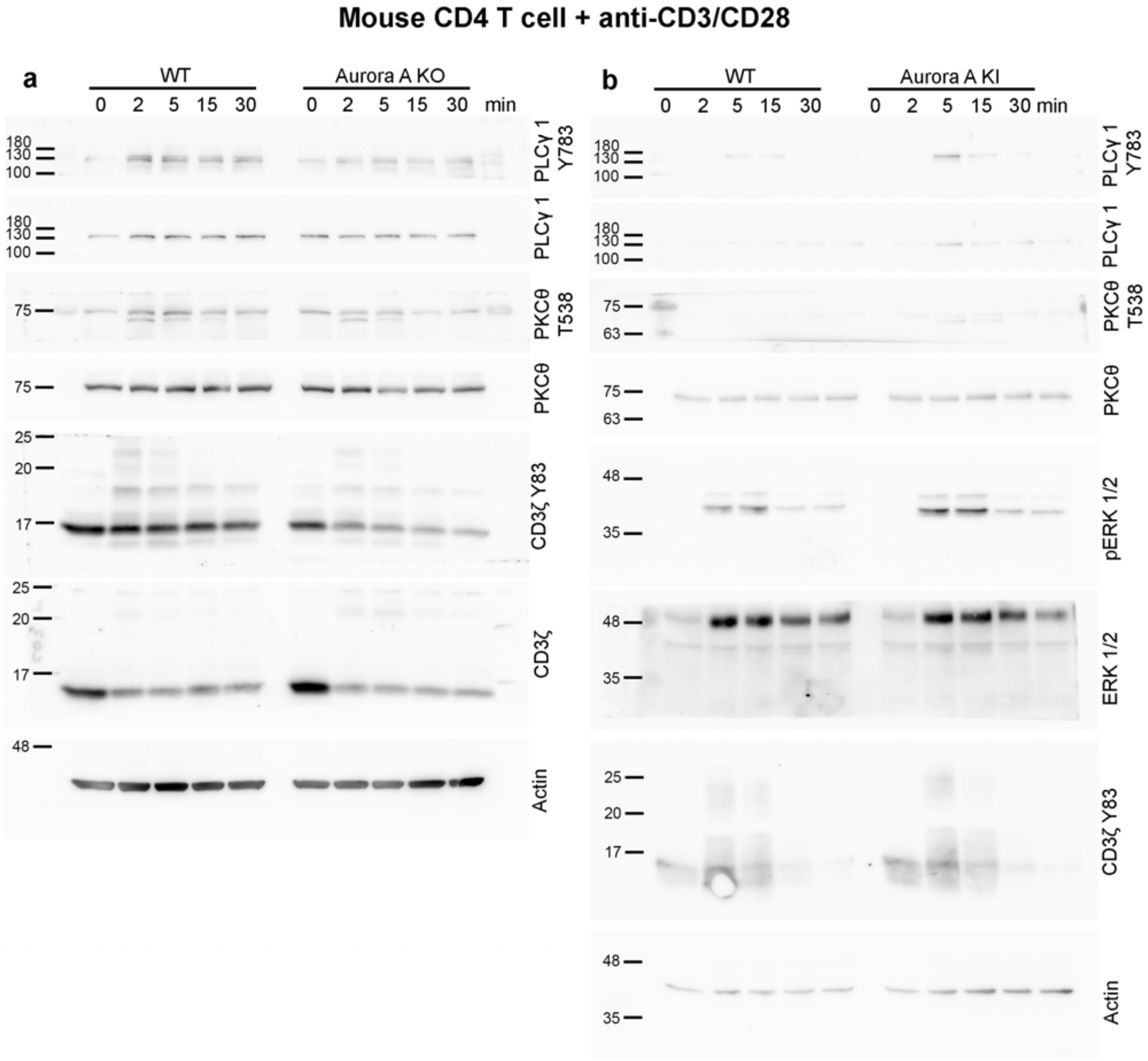

Supplementary Figure 8. Uncropped Western blots. (a) Western blots correspond to Fig. 8b. (**b**) Western blots correspond to Fig. 8e. Molecular weights are indicated.

Suplementary Figure 9

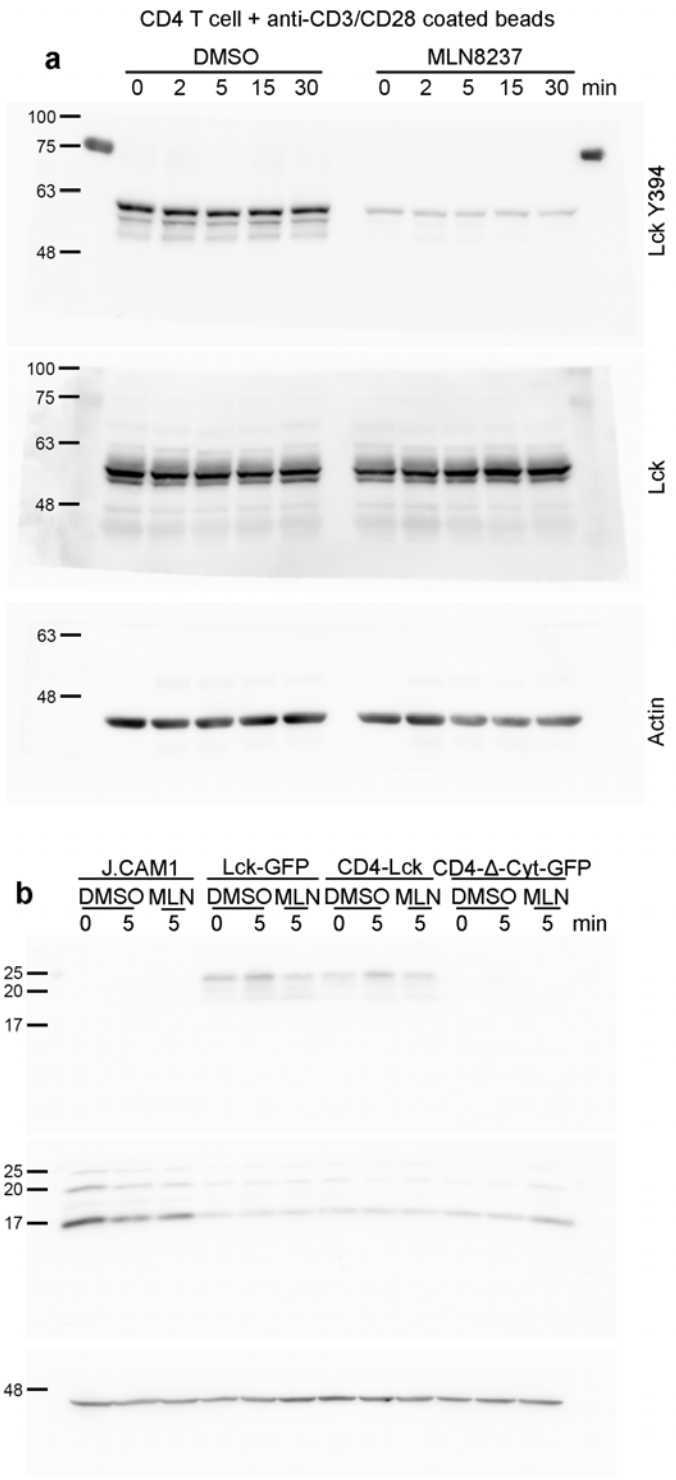

Supplementary Figure 9. Uncropped Western blots. (a) Western blots correspond to Fig. 9c. (**b**) Western blots correspond to Fig 9e. Molecular weights are indicated.

Supplementary Table 1

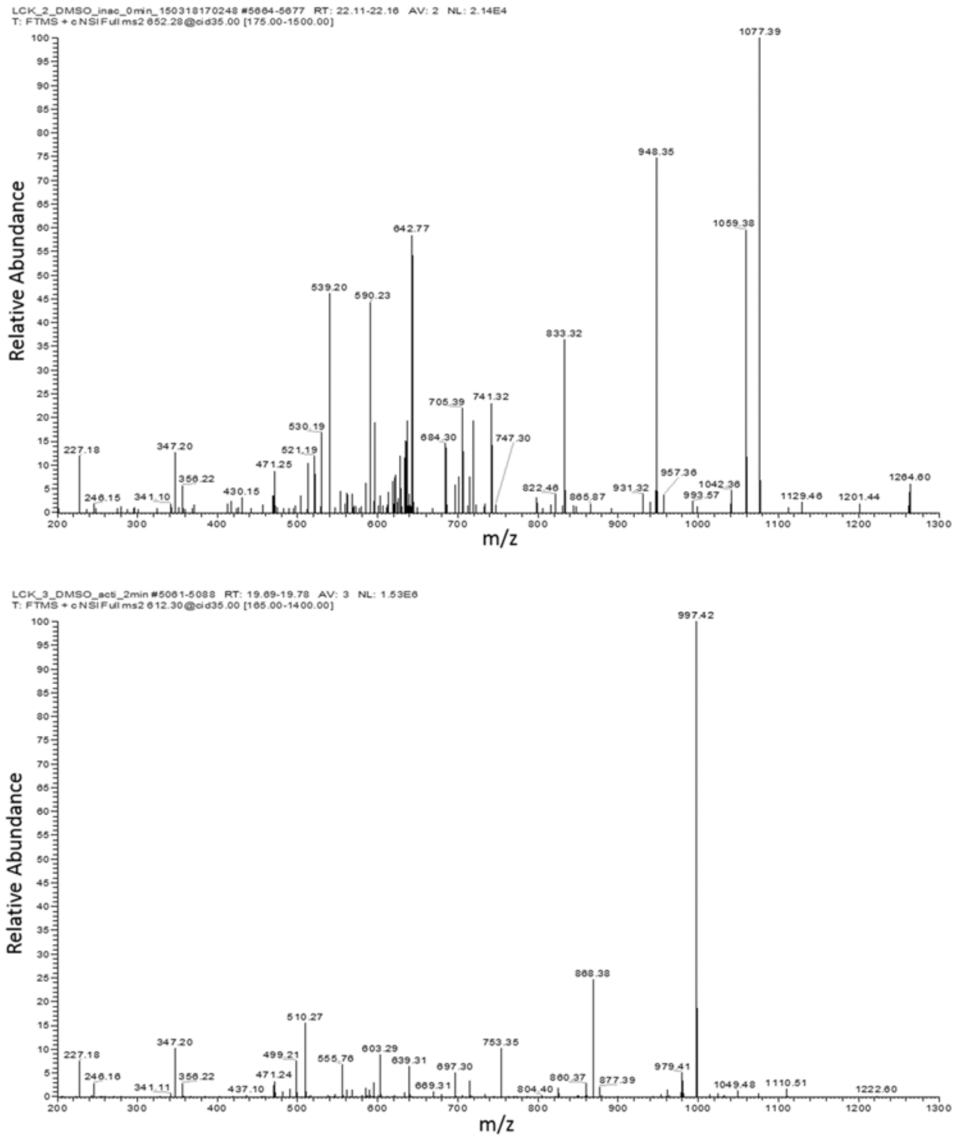

Supplementary Table 1. Examples of MS/MS fragmentation spectra from the Y394-phosphorylated (upper panel) and non-modified (lower panel) forms of Lck peptide LIEDNEYTAR. Ions ascribed to the main *y*- (containing the peptide C-terminus) and *b*- (containing the peptide N-terminus) fragmentation series are indicated. The Y394-phosphorylated and non-modified forms of Lck peptide LIEDNEYTAR were quantified using the MS/MS extracted ion chromatograms of the y8 fragmentation ions.

Supplementary Table 2

Gene	Forward	Reverse
GAPDH	AGCCACATCGCTCAGACAC	GCCCAATACGACCAAATCC
IL-2	AAGTTTTACATGCCCAAGAAGG	AAGTGAAAGTTTTTGCTTTGAGCTA
CD69	CAAGTTCCTGTCCTGTGTGC	GAGAATGTGTATTGGCCTGGA
CD25	CAGCCCCAGCTCATATGCA	TGAGGCTTCTCTTCACCTGGAA

Supplementary Table 2. Sequences of primers used for RT-PCR.
